# A novel monoclonal antibody targeting the hemagglutinin–neuraminidase of peste des petits ruminants virus maintains neutralizing activity by blocking viral adsorption and receptor interaction

**DOI:** 10.1128/jvi.00787-26

**Published:** 2026-06-26

**Authors:** Ruiqi Li, Ashenafi Kiros Wubshet, Shasha Wang, Xiaolong Bai, Lingling Tie, Zaib Ur Rehman, Xiaolong Gao, Huibao Wang, Yu Han, Xiangwei Wang, Xiangping Yin, Yuefeng Sun, Jinxin Xie, Shanhui Ren

**Affiliations:** 1 College of Veterinary Medicine, Xinjiang Agricultural University 117840, Ürümqi, People's Republic of China; 2 State Key Laboratory for Animal Disease Control and Prevention, Lanzhou Veterinary Research Institute, Chinese Academy of Agricultural Sciences 111658, Lanzhou, People's Republic of China; 3 Shandong Binzhou Institute of Animal Husbandry and Veterinary Sciences, Binzhou, People's Republic of China; 4 Department of Poultry Science, Faculty of Veterinary and Animal Sciences, PMAS-Arid Agriculture University, Rawalpindi, Pakistan; 5 College of Agriculture and Animal Husbandry, Qinghai University 207475, Xining, People's Republic of China; 6 School of Environment Engineering, Gansu Forestry Voctech University, Tianshui, People's Republic of China; 7 College of Advanced Agricultural Sciences, Yulin University 233141, Yulin, People's Republic of China; 8 Yazhouwan National Laboratory 626797 , Sanya, People's Republic of China; University of Kentucky College of Medicine, Lexington, Kentucky, USA

**Keywords:** peste des petits ruminants virus, hemagglutinin–neuraminidase, neutralizing antibody, antigenic epitope, viral adsorption, receptor interaction

## Abstract

Peste des petits ruminants (PPR) is a highly contagious viral disease that primarily affects sheep and goats, posing a significant threat to small ruminant livestock production. The protective humoral immunity against Peste des petits ruminants virus (PPRV) is mainly mediated by the hemagglutinin–neuraminidase (HN) glycoprotein. To elucidate the functional antigenic determinants of the PPRV HN protein, we developed a new monoclonal antibody (mAb), HN^1D4-4D9^. Epitope mapping analysis revealed that HN^1D4-4D9^ recognized a novel, conserved epitope (^380^ECLVEACK^387^), including a linear epitope (^381^CLVEACK^387^), and a conformational epitope (^380^ECLVEA^385^) across multiple PPRV genotypes. Alanine-scanning mutagenesis identified key residues within the conserved linear and conformational epitopes of the PPRV HN protein. Surface plasmon resonance analysis demonstrated the high-affinity binding of the identified HN epitope peptide to HN^1D4-4D9^. Using virus pre-treatment and virus-antibody mixture neutralizing assays, HN^1D4-4D9^ exhibited potent neutralizing activity *in vitro*, whereas the post-attachment assay showed that HN^1D4-4D9^ was ineffective after virus binding to cells, underscoring that the antibody acts at the viral adsorption stage. Mechanistically, quantitative real-time PCR and competitive co-immunoprecipitation assays revealed that HN^1D4-4D9^ blocks viral adsorption by preventing HN interaction with the receptors SLAM and nectin-4, rather than by interfering with the fusion protein. Further biochemical assays revealed that several amino acid residues within the novel epitope are critical for the interaction with both receptors. These findings identify a critical neutralizing epitope on PPRV HN, provide mechanistic insights into antibody-mediated viral inhibition, and support the potential application of HN^1D4-4D9^ as a candidate for epitope-based vaccine optimization, development of entry-targeted antiviral drugs, and diagnostic and prophylactic strategies for controlling PPR.

**IMPORTANCE:**

Peste des petits ruminants virus (PPRV) is a highly contagious morbillivirus that causes severe disease in sheep and goats, posing a significant threat to global small ruminant production. Viral entry is initiated by the hemagglutinin–neuraminidase (HN) glycoprotein through its interaction with host receptors. In this study, we identified a novel monoclonal antibody, HN^1D4-4D9^, that targets a highly conserved neutralizing epitope (^380^ECLVEACK^387^) on the PPRV HN protein and exhibits potent *in vitro* antiviral activity. Mechanistically, HN^1D4-4D9^ blocks viral adsorption by preventing HN engagement with the critical cellular receptors SLAM and nectin-4, thereby inhibiting infection at the earliest stage of the viral life cycle. These findings define a key functional neutralizing site on HN and provide essential insights into the molecular basis of PPRV entry and antibody-mediated inhibition. The identified epitope represents a promising target for next-generation epitope-based vaccines and entry-targeted antiviral therapeutics. In addition, HN^1D4-4D9^ may have potential applications in passive immunoprophylaxis, diagnostics, and studies of PPRV pathogenesis.

## INTRODUCTION

Peste des petits ruminants (PPR), also known as goat plague, is a highly contagious viral disease of small ruminants that causes substantial economic losses and threatens livestock production and livelihoods across Africa, the Middle East, Europe, and Asia (1–3). In recent years, PPR has spread into southeastern Europe, including Greece and Romania (4, 5). The World Organization for Animal Health lists PPR as a notifiable animal disease, and in China, it is classified as a Class I Animal Disease. Infection with Peste des petits ruminants virus (PPRV) results in severe systemic disease in sheep and goats characterized by high mortality rates and respiratory and gastrointestinal pathology (1, 2, 4). Despite the existence of four distinct genetic lineages (genotypes I–IV), PPRV constitutes a single serotype; consequently, an effective monovalent vaccine confers broad cross-protection against all circulating strains (2). Currently, live attenuated vaccines based on the Nigeria 75/1 and Sungri 96 strains are widely used for PPR control. However, several limitations, including reduced thermal stability in subtropical regions, constraints in vaccine supply, and incomplete vaccination coverage, have hindered the effectiveness of global eradication efforts (2). Moreover, PPRV infects a broad range of wild ruminants, which often exhibit mild or atypical clinical signs and act as natural reservoirs, facilitating spillover into domestic populations and driving recurrent outbreaks (1). These factors underscore the complexity and long-term challenges in achieving effective and sustainable control of PPR.

PPRV virions are enveloped and pleomorphic, belonging to the order *Mononegavirales*, family *Paramyxoviridae*, subfamily *Paramyxovirinae*, and genus *Morbillivirus* (6). PPRV is genetically related to other important morbilliviruses, including measles virus (MeV), rinderpest virus (RPV), and canine distemper virus (CDV) (6). Similar to other members of the genus *Morbillivirus*, PPRV exhibits dual lymphoid and epithelial cell tropisms (2). The signaling lymphocyte activation molecule family member 1 (SLAMF1, also known as CD150), a membrane glycoprotein expressed on immature cells such as thymocytes, was first identified as the principal receptor mediating morbillivirus entry into lymphoid cells, including PPRV (7). Subsequently, the adherens junction protein nectin-4, also known as poliovirus receptor-like 4 (PVRL4), was identified as an epithelial receptor for MeV (8, 9), CDV (10), and PPRV (11). Nectin-4 is a single-pass transmembrane protein of the immunoglobulin superfamily that is highly expressed in the trachea, placenta, lungs, prostate, stomach, breast, and nasopharynx (12, 13). Together, the identification of SLAM and nectin-4 as cellular receptors for morbillivirus infections has provided critical insights into the pathogenesis, tissue tropism, and mechanisms underlying virus-induced immunosuppression (7, 8, 10, 11).

The genome of PPRV is a non-segmented and negative-sense RNA that encodes six structural proteins, including nucleocapsid (N), phosphoprotein (P), matrix (M), fusion (F), hemagglutinin–neuraminidase (HN), and large polymerase (L), as well as two non-structural proteins, C and V (6). The two envelope glycoproteins, F and HN, play central roles in the early stages of viral infection. Neuraminidase activity has been reported for the PPRV HN (14) and RPV H proteins (14, 15), a feature unusual among members of the genus *Morbillivirus*. Notably, the PPRV HN glycoprotein exhibits a unique combination of hemadsorption and neuraminidase enzyme activities (14). The PPRV HN glycoprotein mediates viral attachment by binding to the cellular receptors SLAM and nectin-4 on susceptible host cells (2). Following receptor engagement, HN activates the F protein, which drives fusion of the viral envelope with the host cell membrane, enabling the viral genome to enter the cytoplasm (2). Similar to the MeV (16), the HN and F glycoproteins of PPRV, particularly HN, are the principal antigenic determinants, eliciting robust humoral immune responses and inducing the production of protective neutralizing antibodies (17, 18).

Vaccination remains the most effective strategy for preventing emerging and re-emerging viral infections in humans and animals (19, 20). In parallel, neutralizing monoclonal antibodies (mAbs) induced by viral infections or generated by hybridoma and recombinant technologies have become powerful tools for the prevention and treatment of viral diseases (21). The protective activity of neutralizing antibodies is mediated not only by direct targeting of viral surface glycoproteins but also by Fc receptor-mediated functions, including antibody-dependent cell-mediated cytotoxicity (ADCC), antibody-dependent cellular phagocytosis (ADCP), as well as complement-dependent cytotoxicity (22–24). For many RNA viruses, cellular entry is initiated by engagement of a single viral surface protein with host receptors, as exemplified by respiratory syncytial virus (RSV) (25, 26), SARS-CoV-2 (27), human immunodeficiency virus type 1 (HIV-1) (28, 29), hepatitis C virus (HCV) (30), and influenza A viruses (IAV) (31, 32). Consistent with this paradigm, several highly potent neutralizing mAbs, such as RSV 199 (25), D25 (26), 7D6/6D6 (27), AP33 (30), VIS410 (32, 33), and C12H5 (31), have been identified, which exert strong antiviral activity by targeting critical viral structural proteins.

The objective of this study was to generate a mAb targeting the PPRV HN glycoprotein to define its functional antigenic determinants and clarify its contribution to viral pathogenesis. Using conventional hybridoma technology, we produced a mAb, designated HN^1D4-4D9^, against the PPRV HN protein. We systematically identified and characterized a novel, highly conserved linear and conformational neutralizing epitope on HN across multiple PPRV genotypes. Crucially, HN^1D4-4D9^ exhibited potent neutralizing activity *in vitro*. Mechanistic analyses demonstrated that this antibody inhibits infection at the earliest stage by blocking viral adsorption, primarily through steric interference with HN interactions with the cellular receptors SLAM and nectin-4. Identification of this critical neutralizing epitope provides a rational foundation for the development of epitope-based antiviral strategies and next-generation vaccine candidates. In addition, HN^1D4-4D9^ represents a valuable tool for PPR prophylaxis, diagnostic development, and mechanistic studies of PPRV pathogenesis.

## RESULTS

### Epitope mapping of the PPRV HN protein recognized by a monoclonal antibody under native and denaturing conditions

To generate a monoclonal antibody against the PPRV HN protein, BALB/c mice were immunized with a truncated PPRV HN protein (amino acids 223-551). Hybridoma technology was employed, and culture supernatants were screened for reactivity with the truncated HN protein by enzyme-linked immunosorbent assay (ELISA) (Fig. S1C). After multiple rounds of screening and subcloning, one hybridoma clone (1D4-4D9), designated HN^1D4-4D9^, exhibited exceptionally strong binding to the PPRV HN protein and was selected for further characterization (Fig. S1D). Isotyping analysis revealed that HN^1D4-4D9^ belonged to the IgG2b subclass, and the purified antibody concentration was 7.5 mg/mL.

**Fig 1:**
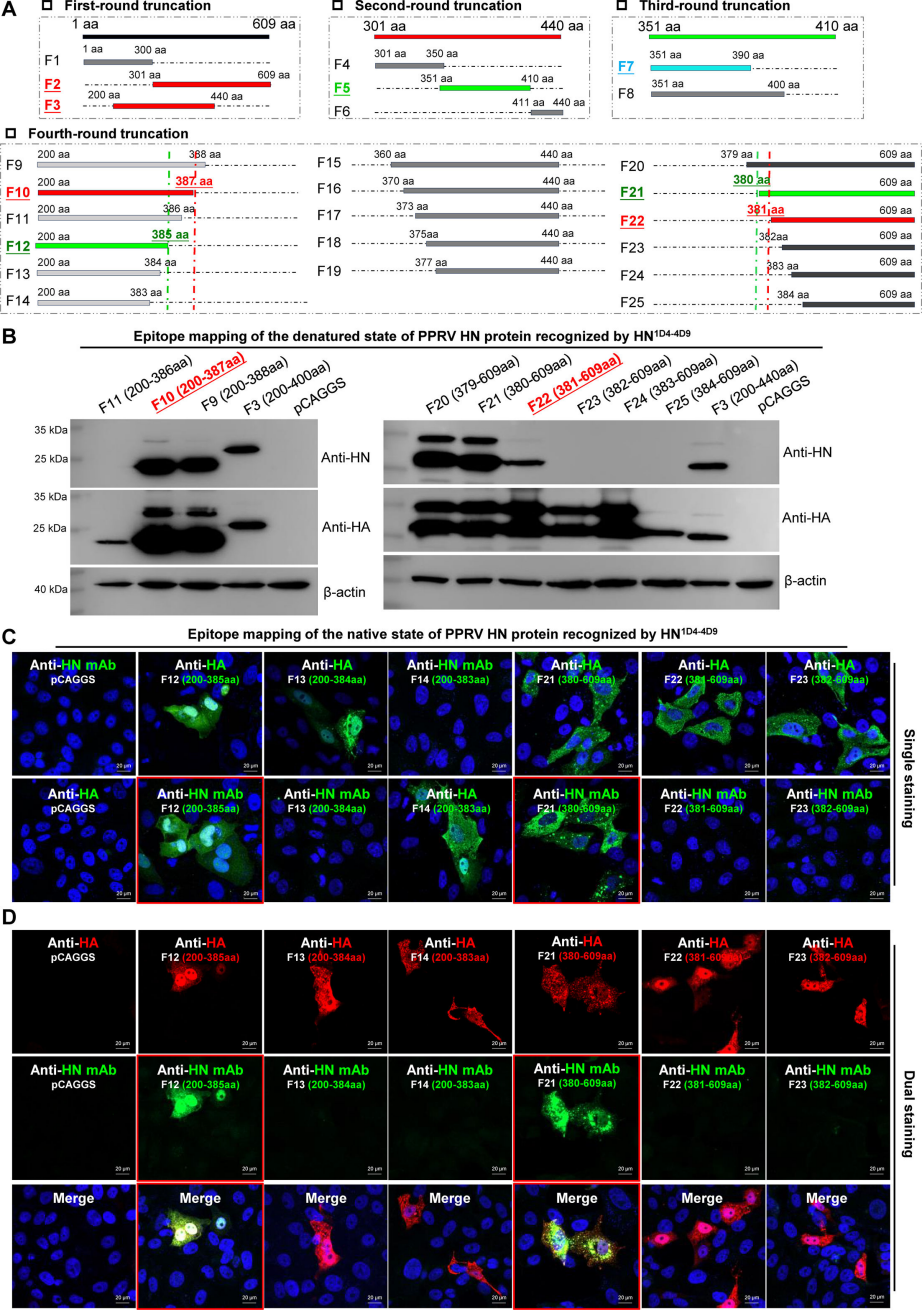
Epitope mapping of the native and denatured states of the PPRV HN protein recognized by HN^1D4-4D9^. (**A**) Schematic representation of plasmid construction across four rounds of truncation. Different colors and underlining of the F2, F3, F5, F7, F10, F12, F21, and F22 fragments indicate the positive groups for mapping linear and conformational epitopes in the first, second, third, and fourth rounds of truncation. (**B**) WB analysis of representative HN fragments for linear epitope mapping. Plasmids, including pCAGGS, F3-pCAGGS (200–440aa)-HA, F9-pCAGGS (200–388aa)-HA, F10-pCAGGS (200–387aa)-HA, F11-pCAGGS (200–386aa)-HA, F20-pCAGGS (379–609aa)-HA, F21-pCAGGS (380–609aa)-HA, F22-pCAGGS (381–609aa)-HA, F23-pCAGGS (382–609aa)-HA, F24-pCAGGS (383–609aa)-HA, and F25-pCAGGS (384–609aa)-HA, were transfected into 293T cells. After 24 h post-transfection, the cell samples were harvested for WB analysis. The virulent PPRV strain is abbreviated as V. (**C**) IFA analysis of six key HN fragments for conformational epitope mapping using single staining. Plasmids, including pCAGGS, F12-pCAGGS (200–385aa)-HA, F13-pCAGGS (200–384aa)-HA, F14-pCAGGS (200–383aa)-HA, F21-pCAGGS (380–609aa)-HA, F22-pCAGGS (381–609aa)-HA, and F23-pCAGGS (382–609aa)-HA, were transfected into Vero cells. After 24 h post-transfection, the cell samples were prepared for IFA analysis. The virulent PPRV strain is abbreviated as V. The red square border for the F12 and F21 fragments indicates the positive group for mapping conformational epitopes in the first, second, third, and fourth truncation rounds. (**D**) IFA analysis of six key HN fragments for conformational epitope mapping using dual staining. Plasmids, including pCAGGS, F12-pCAGGS (200–385aa)-HA, F13-pCAGGS (200–384aa)-HA, F14-pCAGGS (200–383aa)-HA, F21-pCAGGS (380–609aa)-HA, F22-pCAGGS (381–609aa)-HA, and F23-pCAGGS (382–609aa)-HA, were transfected into 293T cells. After 24 h post-transfection, the cell samples were prepared for IFA analysis. The virulent PPRV strain is abbreviated as V.

To map the linear and conformational antigenic epitopes recognized by HN^1D4-4D9^, antigenic regions of the full-length PPRV HN protein were first predicted using the IEDB online analysis tool (Fig. S2A). To localize the epitope, the first round of truncated HN fragments (1–300, 200–440, and 301–609 aa) was constructed (Table S1) and analyzed by Western blot (WB) and indirect immunofluorescence assay (IFA). These WB and IFA analyses indicated that both linear and conformational epitopes recognized by HN^1D4-4D9^ were located within residues 301–440 (Fig. S2B and Fig. S3A). To further refine this region, a second round of truncated fragments (301–350, 351–410, and 411–440 aa) was expressed (Fig. 1A and Fig. S2C). WB and IFA results localized the epitope to residues 351–410 (Fig. S2D and Fig. S3B). This region was subsequently subdivided in the third round of truncation into two overlapping fragments (351–390 and 351–400 aa), and analysis revealed that the epitope was confined to residues 351–390 (Fig. 1A and Fig. S2E and Fig. S3C). Fine epitope mapping was performed using successive single amino acid truncations at both the N- and C-termini during the fourth round of truncation (Fig. 1A). As shown in Fig. 1B and Fig. S3D and E, WB analysis identified the linear epitope spanning residues 381–387. Notably, the protein signal intensity of fragment F22 (381–609 aa) was significantly weaker than that of F21 (380–609 aa), suggesting that amino acid residue 380 is also critical for HN^1D4-4D9^ recognition under denaturing conditions (Fig. 1B). In contrast, single- and dual-staining IFA data defined the conformational epitope as spanning residues 380–385 (Fig. 1C and D and Fig. S2E). Finally, stepwise truncation and overlapping expression identified a partially overlapping linear epitope (amino acids 381–387) under denaturing conditions and a conformational epitope (amino acids 380–385) recognized in the native state of the PPRV HN protein. Collectively, these fine-mapping analyses defined ECLVEACK as a critical antigenic determinant of PPRV HN, representing a novel epitope spanning amino acid residues 380–387.

### Sequence alignment, epitope verification, and binding affinity analysis of HN^1D4-4D9^-recognized epitope peptides

**Fig 2:**
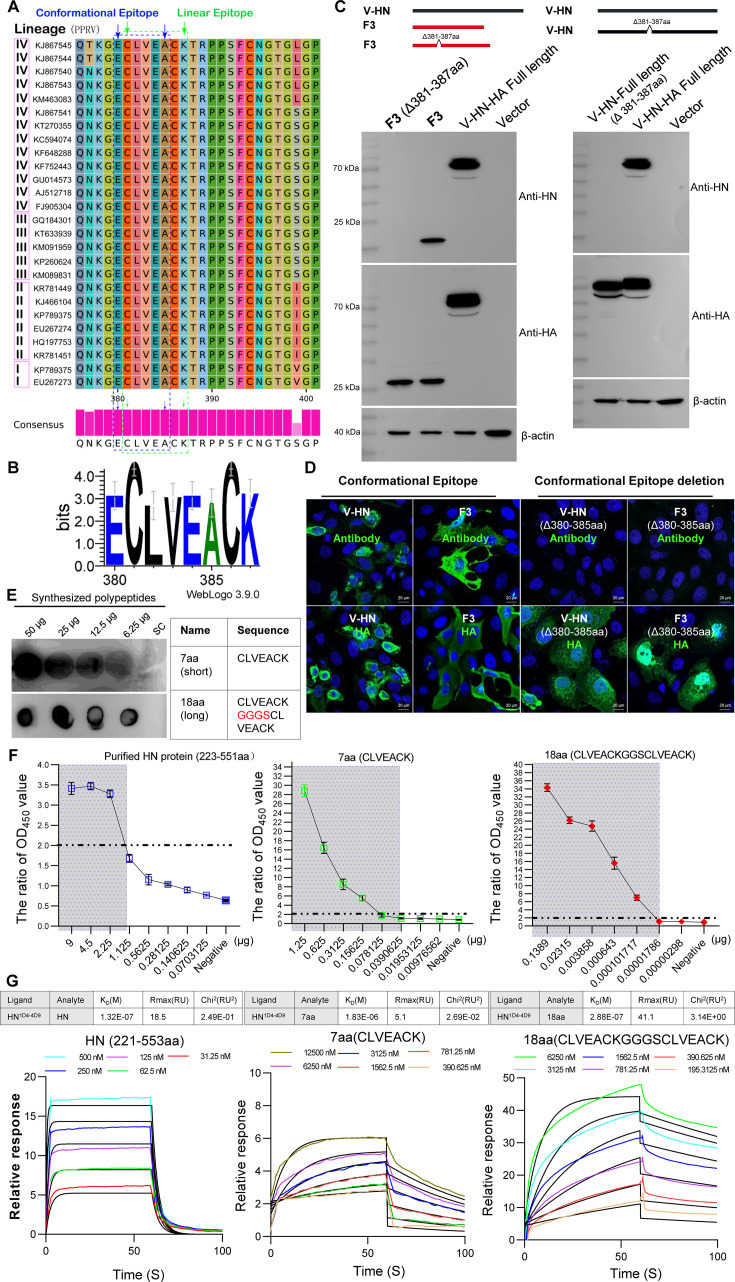
Sequence alignment and confirmation of a novel antigenic epitope recognized by HN^1D4-4D9^. (**A**) Amino acid sequence alignment of the PPRV HN protein. The sequences were downloaded from GenBank and analyzed using DNASTAR and Jalview. Protein sequences were aligned using MUSCLE with the following display parameters: the “Flower” color scheme, gridlines, and consensus sequence. (**B**) Diversity of residues at each position in the linear and conformational epitope sequences. A graphical representation of the amino acid sequence from positions 380–387 was generated using WebLogo 3. Each logo displays stacks of symbols with one stack per position in the sequence. All parameters were set to default. (**C**) WB analysis of truncated PPRV HN plasmids. The plasmids, including pCAGGS, F3-pCAGGS HN(Δ381–387aa)-HA, F3-pCAGGS HN(200–440aa)-HA, pCAGGS-V-HN(V-HN)(Δ381–387aa)-HA, and pCAGGS-V-full-length HN(V-HN)-HA, were transfected into 293T cells. After 24 h post-transfection, the cells were prepared for WB analysis. The virulent PPRV strain is abbreviated as V. (**D**) IFA analysis of truncated PPRV HN plasmids. The plasmids, including F3-pCAGGS HN(200–440aa)-HA, F3-pCAGGS HN(Δ380–385aa)-HA, pCAGGS V-HN-HA, and pCAGGS V-HN(Δ380–385aa)-HA, were transfected into Vero cells. After 24 h post-transfection, the cells were prepared for IFA analysis. The virulent PPRV strain is abbreviated as V. (**E**) Dot blot analysis of synthetic polypeptides recognized by HN^1D4-4D9^. Synthetic polypeptides (CLVEACK and CLVEACKGGGSCLVEACK) were serially diluted (2×) to 50, 25, 12.5, and 6.25 μg/mL and used as the reactive antigens. The scrambled polypeptide control is abbreviated as SC. (**F**) Indirect ELISA analysis of the purified HN protein and the synthetic polypeptides against HN^1D4-4D9^. The purified HN protein was serially diluted (2×) to 9, 4.5, 2.25, 1.125, 0.5625, 0.28125, 0.140625, and 0.0703125 μg/μL. The single-epitope polypeptides (CLVEACK) were serially diluted (2×) to 1.25, 0.625, 0.3125, 0.15625, 0.078125, 0.0390625, 0.01953125, and 0.00976562 μg. The tandem epitope polypeptides (CLVEACKGGGSCLVEACK) were serially diluted (2×) to 0.1389, 0.02315, 0.003858, 0.000643, 0.000101717, 0.00001786, and 0.00000298 μg/μL. (**G**) Surface plasmon resonance (SPR) kinetics and affinity measurements of HN^1D4-4D9^ binding to the purified HN protein and synthetic epitope peptides. The binding affinity and kinetics were measured using a Biacore K8 instrument. The purified HN protein was serially diluted (2×) to 500, 250, 125, 62.5, and 31.25 nM. The single-epitope polypeptides (CLVEACK) were serially diluted (2×) to 12,500, 6,250, 3,125, 1,562.5, 781.25, and 390.325 nM concentrations. The tandem epitope polypeptides (CLVEACKGGGSCLVEACK) were serially diluted (2×) to 6,250, 3,125, 1,562.5, 781.25, 390.625, and 195.3125 nM concentrations, with each curve representing the biosensor response at each concentration.

To determine whether the newly identified linear and conformational epitopes were conserved across PPRV genotypes, HN amino acid sequences from 26 representative PPRV strains were subjected to multiple sequence alignments. As shown in Fig. 2A and B, both multiple-sequence alignment and WebLogo analyses revealed that the linear epitope (^381^CLVEACK^387^) and the conformational epitope (^380^ECLVEA^385^) were highly conserved across four PPRV genotypes (I–IV).

To further validate the identified antigenic determinants, epitope-deletion mutant plasmids, including F3 (Δ381–387), F3 (Δ380–385), V-HN (Δ381–387), and V-HN (Δ380–385), were constructed using specific primers (Table S1). WB analysis showed that, in contrast to the positive controls (F3 and V-HN) and the vector control, HN^1D4-4D9^ failed to recognize the epitope-deleted proteins (Fig. 2C). IFA further confirmed the specificity of HN^1D4-4D9^ for binding to the conformational epitope (^380^ECLVEA^385^) in comparison with an HA-tag positive control (Fig. 2D). These WB and IFA results verified the accuracy of the identified contiguous linear and conformational epitopes. To further confirm the sequence accuracy of the determinant epitope, synthetic polypeptides corresponding to the single epitope (CLVEACK) and the tandem repeat epitope (CLVEACKGGGSCLVEACK) were generated. As shown in Fig. 2E and F, dot blot and indirect ELISA analyses demonstrated a dose-dependent binding of the purified HN protein and both epitope peptides to HN^1D4-4D9^, as compared with the negative control. Notably, the tandem epitope peptide exhibited greater sensitivity than the single-epitope peptides. SPR analysis was subsequently performed to quantify the binding affinity of HN^1D4-4D9^ to the purified HN protein and the synthetic epitope peptides. The apparent equilibrium dissociation constants (*K*_D_) for the interactions were determined using a kinetic fitting affinity model, yielding values of 2.88 × 10⁻⁷, 1.32 × 10⁻⁷, and 1.83 × 10⁻⁶ for the tandem epitope peptide, the purified HN protein, and the single-epitope peptide, respectively (Fig. 2G). The *K*_D_ values derived from the SPR analysis indicated that the tandem epitope peptide exhibited higher affinity than the single-epitope peptide, suggesting a robust, specific interaction with potential for targeted applications. Collectively, these results confirm the accuracy of linear epitope mapping and demonstrate the strong and specific binding of HN^1D4-4D9^ to the identified epitopes.

### Identification of critical residues within linear and conformational epitopes recognized by HN^1D4-4D9^

**Fig 3:**
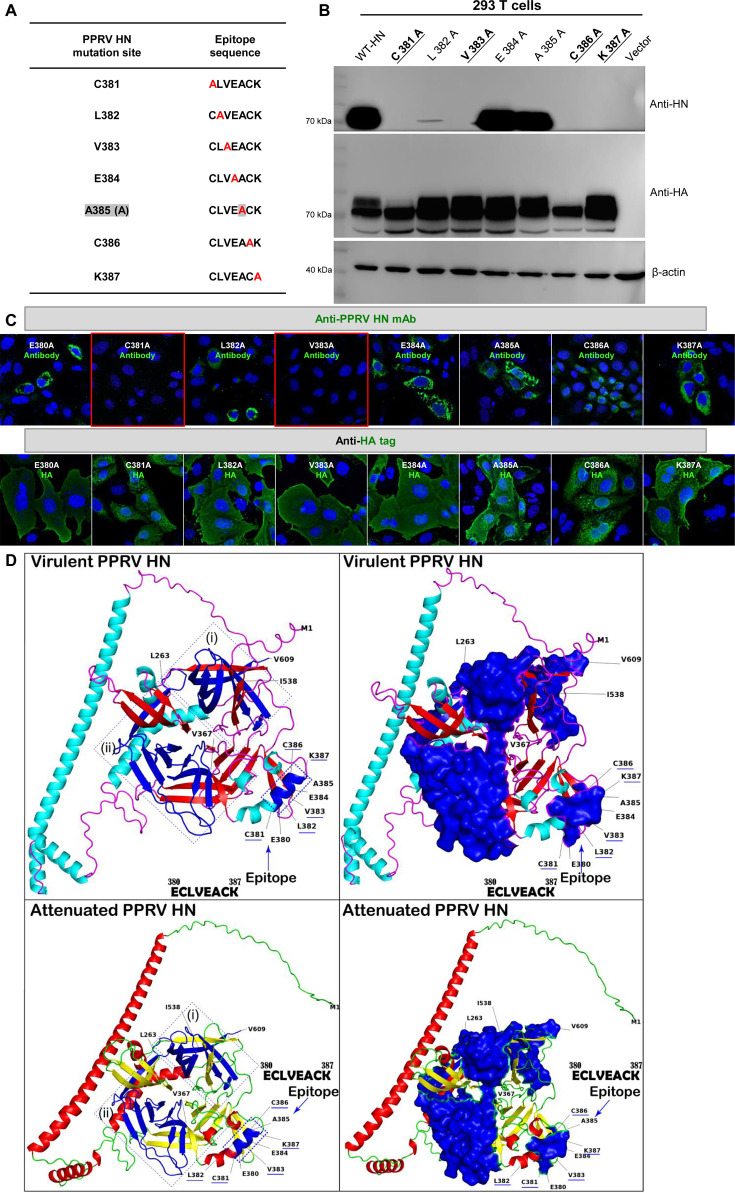
Identification and verification of the critical amino acid sites within this conserved continuous linear and conformational epitope of the PPRV HN protein. (**A**) Alanine-scanning mutagenesis of the linear and conformational epitopes of PPRV HN. The amino acid residues in the PPRV HN epitope were mutated to alanine residues. Alanine (A) substitutions are shown in red. A385(A) indicates a synonymous mutation in the HN gene. (**B**) WB analysis of PPRV HN mutant plasmids recognized by HN^1D4-4D9^. 293T cells were transfected with pCAGGS-HN-HA, pCAGGS-HN(C381A)-HA, pCAGGS-HN(L382A)-HA, pCAGGS-HN(V383A)-HA, pCAGGS-HN(E384A)-HA, pCAGGS-HN(A385A)-HA, pCAGGS-HN(C386A)-HA, pCAGGS-HN (K387A)-HA, or pCAGGS plasmids. After 24 h post-infection, 293T cells were collected and analyzed via WB. (**C**) IFA analysis of PPRV HN mutant plasmids recognized by HN^1D4-4D9^. Vero cells were transfected with pCAGGS-HN(C381A)-HA, pCAGGS-HN(L382A)-HA, pCAGGS-HN(V383A)-HA, pCAGGS-HN(E384A)-HA, pCAGGS-HN(A385A)-HA, pCAGGS-HN(C386A)-HA, pCAGGS-HN(K387A)-HA, or pCAGGS plasmids. After 24 h post-infection, Vero cells were collected, fixed, and visualized via IFA. HA tag and HN^1D4-4D9^ (green); nuclei (blue). (**D**) Spatial structural simulation and prediction of HN proteins from virulent and attenuated PPRV. The nucleotide sequences of virulent and attenuated PPRV HN were translated into their corresponding amino acid sequences. These sequences were submitted to the AlphaFold Protein Structure Database and saved in PDB format. Subsequently, structure simulation and visualization were performed using the open-source PyMOL software. Symbol (i) indicates the previously dominant antigenic domain (538–609 aa); symbol (ii) indicates the previously dominant antigenic domain (263–368 aa). The underlined text indicates the key amino acid positions for linear and conformational epitopes.

To identify key amino acid residues within the conserved linear and conformational epitopes recognized by HN^1D4-4D9^, alanine-scanning mutagenesis was performed (Fig. 3A). WB analysis using an HA-tag antibody as a positive control confirmed successful expression of all HN mutant plasmids (Fig. 3B and Table S2). Compared with the wild-type HN (WT-HN) and the synonymous control mutation (A385A), alanine substitutions revealed that four residues (C381, V383, C386, and K387) are essential for the recognition of the antigen-antibody complex, as these mutations abolished the reactivity of the HN protein with HN^1D4-4D9^. In contrast, the L382A mutation reduced but did not eliminate antibody binding, indicating a contributory but non-essential role in epitope recognition. Consistent with these findings, IFA further demonstrated that, relative to the HA-tag control and the A385A synonymous mutant, substitutions at C381 and V383 completely abolished the binding of HN^1D4-4D9^ to the conformational epitope of the HN protein (Fig. 3C, red box), indicating that C381 and V383 are the core residues required for the conformational epitope recognition.

To further define the structural context of the critical residues, the HN structures of both virulent and attenuated PPRV strains were predicted using AlphaFold and visualized with PyMOL. Notably, all residues (E380, C381, L382, V383, E384, A385, C386, and K387) within the identified epitope region of the HN protein were localized to an alpha-helix in the 3D structural simulation (Fig. 3D and Fig. S4). Structural analysis revealed that this continuous epitope motif (380–387 aa) occupies a spatially distinct region from two known immunodominant, discontinuous neutralizing site residues (263–368 and 538–609 aa) on HN (Fig. 3D).

### Potent *in vitro* neutralizing activity of HN^1D4-4D9^ against PPRV in a Vero-SN cell model

As shown in the flowchart in Fig. S5A, using a lentiviral packaging system, we engineered a Vero cell line that stably expressed sheep-derived SLAM and nectin-4 genes. Following multiple rounds of puromycin and blasticidin drug selection and subclonal screening, a stable cell line, designated as Vero-SN, was established. As shown in Fig. S5B, IFA analysis confirmed that both SLAM and nectin-4 were stably expressed and localized to the cell membrane. This dual-receptor Vero-SN cell line stably co-expresses SLAM and nectin-4, providing a standardized, reproducible platform for studying viral pathogenesis and for performing neutralizing antibody assays under identical receptor conditions. Subsequently, viral infection experiments confirmed that the attenuated PPRV (Nigeria 75/1 strain) replicated efficiently in Vero-SN cells (Fig. S5C and D) and induced typical syncytium formation (Fig. S5E). The attenuated PPRV exhibited extensive syncytia formation and higher intracellular and extracellular viral titers in Vero-SN cells than in wild-type Vero cells (Fig. S6), indicating the successful establishment of a robust *in vitro* PPRV infection model.

**Fig 4:**
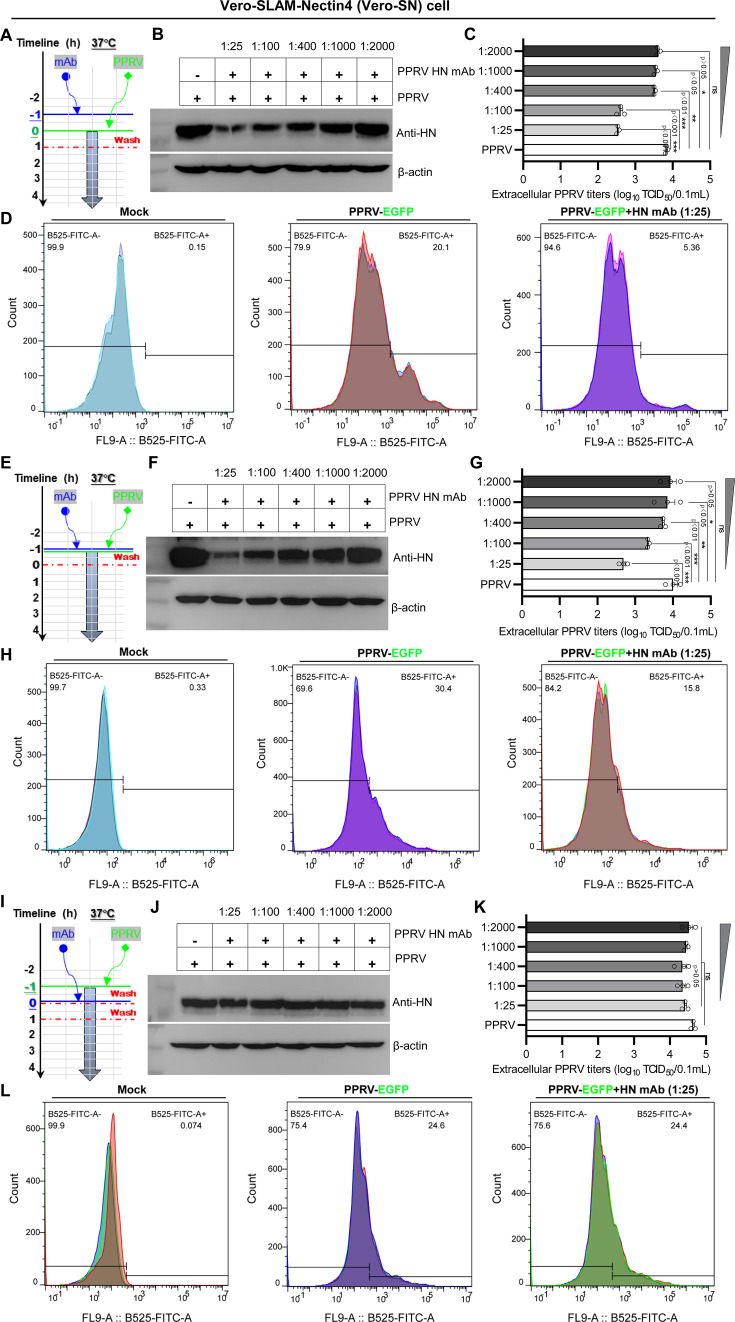
Identification of potent neutralizing activity of HN^1D4-4D9^ against PPRV in Vero-SN. (**A**, **E**, and **I**) Schematic timeline of the neutralization assay used to evaluate HN^1D4-4D9^ against PPRV infection. The diagram illustrates the experimental workflow over time (y-axis) for the three HN^1D4-4D9^ treatment strategies in response to PPRV infection: pre-, post-, and simultaneous mixture treatments. Key procedural steps are color-coded. Blue: Treatment of Vero-SN cells or virus with HN^1D4-4D9^; green: exposure to extracellular mature PPRV virions (Nigeria 75/1 strain or EGFP-PPRV); red: critical wash with PBS to remove unbound, non-internalized virions after viral adsorption. (**B**, **F**, and **J**) WB analysis of total HN protein expression under HN^1D4-4D9^ antibody pre-treatment, virus–antibody mixture treatment, or post-treatment conditions. Vero-SN cells were infected with the Nigeria 75/1 strain (TCID_50_ = 10^4.806^) at an MOI of 1. After 72 h post-infection, cell samples were collected and analyzed by WB. (**C**, **G**, and **K**) 50% tissue culture infectious dose (TCID_50_) determination of antibody-mediated viral infectivity under antibody HN^1D4-4D9^ pre-treatment, virus–antibody mixture treatment, or post-treatment conditions. Vero-SN cells were infected with the Nigeria 75/1 strain (TCID_50_ = 10^4.806^) at an MOI of 1. After 72 h post-infection, cell supernatant samples were collected, and TCID_50_ was calculated using the Reed-Muench method. Statistical significance is denoted as ns (*P* > 0.05), *P < 0.05, **P < 0.01, and ***P < 0.001. Data from three independent experiments are presented as mean ± SEM. (**D**, **H**, and **L**) Flow cytometry analysis of the percentage of EGFP-positive-infected cells under HN^1D4-4D9^ pre-treatment, virus–antibody mixture treatment, or post-treatment conditions against EGFP-PPRV. Vero-SN cells were infected with EGFP-PPRV (TCID_50_ = 10^4.614^) at an MOI of 1. After 48 h post-infection, cells were collected and analyzed by flow cytometry.

In this study, the neutralizing activity of HN^1D4-4D9^ against attenuated PPRV was subsequently evaluated in the Vero-SN cell model using three complementary experimental strategies. In the antibody pre-treatment assay, Vero-SN cells were incubated with HN^1D4-4D9^ for 1 h prior to infection with either PPRV (Nigeria 75/1 strain) or EGFP-expressing PPRV (Fig. 4A and Fig. S8A). WB and TCID_50_ assays demonstrated that HN^1D4-4D9^ effectively inhibited the total mount HN expression of the intracellular PPRV and reduced the antibody-mediated extracellular viral infectivity in a dose-dependent manner (Fig. 4B and C), which was consistent with the marked reduction in EGFP fluorescence intensity and syncytium formation (Fig. S7A). Flow cytometry further confirmed a significant fraction decrease in the percentage of infected cells following HN^1D4-4D9^ pre-treatment (Fig. 4D). In the virus–antibody mixture assay (Fig. 4E), HN^1D4-4D9^ was incubated with attenuated PPRV or EGFP-PPRV for 1 h at 37°C prior to infection. As shown in Fig. 4F and G, the total mount HN expression and the antibody-mediated infectivity reduction of the antibody mixture group were significantly lower than those in the PPRV-positive group, indicating that HN^1D4-4D9^ retained potent inhibitory activity against PPRV in a concentration-dependent manner, which is consistent with the reduced EGFP signal and syncytium formation (Fig. S7B). Flow cytometric analysis further confirmed the strong inhibitory effect of HN^1D4-4D9^ on the fraction of EGFP-PPRV infected cells (Fig. 4H). Collectively, these results demonstrate that HN^1D4-4D9^ exhibits potent *in vitro* neutralizing activity against PPRV under both antibody pre-treatment and virus–antibody mixture conditions.

To further assess the neutralizing activity of HN^1D4-4D9^ during the early stages of PPRV replication, a post-attachment assay was performed in Vero-SN cells. Cells were first infected with PPRV or EGFP-PPRV for 1 h to allow viral binding, followed by incubation with HN^1D4-4D9^ for an additional 1 h (Fig. 4I). As shown in Fig. 4J and K, the HN expression and the antibody-mediated reduction in the infection rate showed no significant difference between the antibody treatment and PPRV-infected groups, suggesting that HN^1D4-4D9^ failed to effectively inhibit both intracellular and extracellular viral replication under these conditions, which is consistent with the unchanged EGFP fluorescence intensity and syncytium formation observed in Fig. S7C. Flow cytometric analysis confirmed that post-attachment treatment with HN^1D4-4D9^ did not reduce the productive fraction of infectious EGFP-PPRV cells (Fig. 4L). Collectively, these post-attachment assays demonstrated that HN^1D4-4D9^ lacks neutralizing activity after viral binding, indicating that its antiviral effect is primarily exerted at the viral entry stage rather than at the post-entry steps of the replication cycle.

### HN^1D4-4D9^ neutralizes PPRV by blocking viral adsorption and HN-receptor interactions

**Fig 5:**
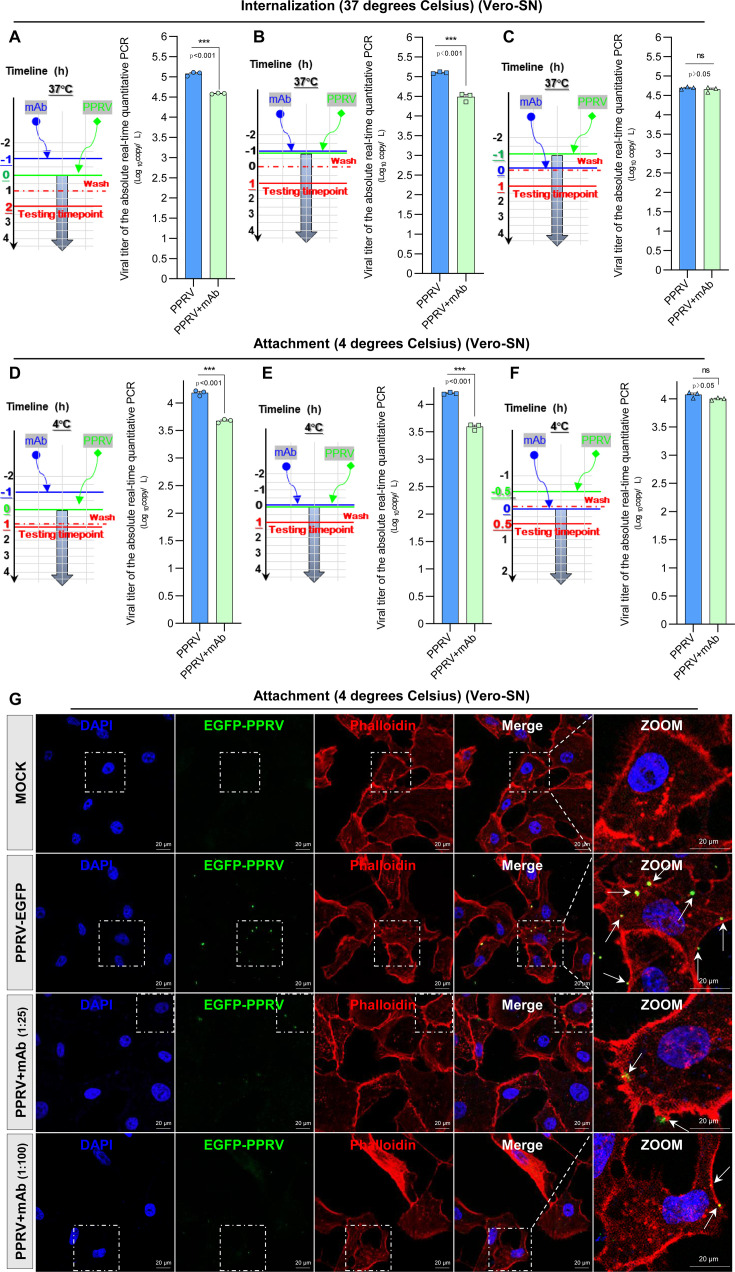
HN^1D4-4D9^ maintains its neutralizing activity by inhibiting viral attachment to host cells. (**A**, **B**, and **C**) qPCR detection of the viral genome under HN^1D4-4D9^ pre-, post-, or simultaneous mixture treatment prior to infection. The diagram illustrates the internalization experimental workflow over time (y-axis) for pre-, post-, and simultaneous mixture treatments of HN^1D4-4D9^ in response to PPRV virions (Nigeria 75/1, TCID_50_ = 10^4.806^) at an MOI of 1 at 37°C. After 2 h of PPRV infection, Vero-SN cell samples were collected for qPCR analysis at the time point of testing. These viral genomes were extracted, reverse-transcribed, and detected. Statistical significance is denoted as: ns (*P* > 0.05), *P < 0.05, **P < 0.01, and ***P < 0.001. Data from three independent experiments are presented as mean ± SEM. (**D**, **E**, and **F**) qPCR detection of the viral genome under HN^1D4-4D9^ pre-, post-, or simultaneous mixture treatment prior to infection. The diagram illustrates the attachment experimental workflow over time (y-axis) for pre-, post-, and simultaneous mixture treatments of HN^1D4-4D9^ in response to PPRV virions (Nigeria 75/1, TCID_50_ = 10^4.806^) at an MOI of 1 at 4°C. After 1 h of PPRV infection, Vero-SN cell samples were collected for qPCR analysis at the time point of testing. Statistical significance is denoted as ns (*P* > 0.05), *P < 0.05, **P < 0.01, and ***P < 0.001. Data from three independent experiments are presented as mean ± SEM. (**G**) IFA analysis of viral adsorption under HN^1D4-4D9^ simultaneous mixture treatment against EGFP-PPRV. Vero-SN cells were seeded into 12-well plates and pre-chilled for 45 min at 4°C. These pre-chilled Vero-SN cells were inoculated with EGFP-PPRV virions (TCID_50_ = 10^4.614^) at an MOI of 1 or with a simultaneous mixture treatment with HN^1D4-4D9^ (1:25 or 1:100) at an MOI of 1 for 1 h at 4°C. After viral adsorption, Vero-SN cells were collected, fixed, and visualized via IFA. EGFP-PPRV (green); phalloidin (red); nuclei (blue).

To elucidate the mechanism of HN^1D4-4D9^ neutralization, we performed viral adsorption and internalization inhibition assays. As shown in Fig. 5A and B, under conditions permissive for both attachment and entry (37°C), pre-treatment of Vero-SN cells with HN^1D4-4D9^ for 1 h, or pre-incubation of the virus–antibody mixture significantly reduced PPRV genomic RNA levels, as quantified by quantitative PCR (qPCR). In contrast, post-treatment with HN^1D4-4D9^ for 1 h after PPRV infection did not affect viral RNA copy numbers (Fig. 5C). These results demonstrate that HN^1D4-4D9^ neutralizes PPRV by blocking an early stage of infection, either prior to or during viral internalization.

To further determine whether HN^1D4-4D9^ interferes with the initial viral adsorption step, Vero-SN cells were incubated with PPRV in the presence of the antibody at 4°C, a temperature that permits viral binding but not entry. Under both antibody pre-treatment and mixture conditions for 1 h, qPCR analysis revealed a marked reduction in cell-associated viral RNA (Fig. 5D and E). In contrast, in the post-attachment assay, where cells were first incubated with PPRV at 4°C for 0.5 h to allow binding, and antibodies were added after removing unbound virus, viral RNA levels were comparable to those of the untreated control (Fig. 5F). Subsequently, viral adsorption was further evaluated by IFA. Pre-chilled Vero-SN cells were incubated for 1 h at 4°C with EGFP-PPRV alone or co-treated with HN^1D4-4D9^ antibody (1:25 or 1:100). Relative to the virus-only control, HN^1D4-4D9^ treatment resulted in a pronounced reduction of cell-bound EGFP fluorescence, demonstrating significant inhibition of viral adsorption (Fig. 5G). These findings demonstrate that HN^1D4-4D9^ directly blocks virion attachment to the host cell surface and fails to exert neutralizing activity after viral adsorption, indicating that its antiviral effect is primarily exerted at the initial adsorption stage of infection.

**Fig 6:**
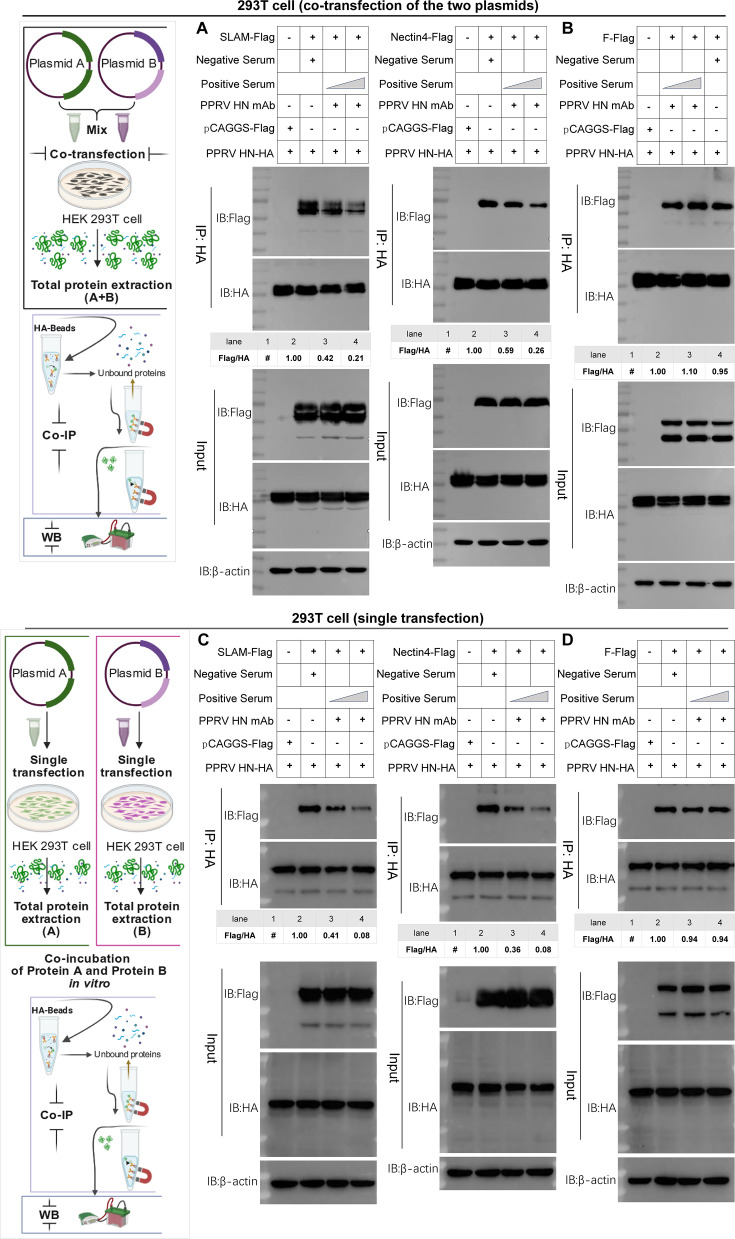
HN^1D4-4D9^ maintains its neutralizing activity by blocking the interaction between the HN protein and its receptors. (**A **and **B**) Competitive co-immunoprecipitation (co-IP) analysis of protein–protein interactions between HN and SLAM or nectin-4 under co-transfection conditions. pCAGGS-flag (5 µg), pCAGGS-sheep-SLAM-Flag (5 µg), pCAGGS-sheep-nectin-4-Flag (5 µg), and pCAGGS-virulent PPRV F(V-F)-Flag were co-transfected with pCAGGS-Virulent-PPRV HN (V-HN)-HA (5 µg) into 293T cells in a 100 cm dish. After 24 h post-transfection, cell samples were collected for Co-IP analysis. The cell lysates were evenly divided into four aliquots, and additional negative mouse antibody (IgG) and purified HN^1D4-4D9^ (1:25 and 1:100) were added, followed by an equal volume of immunomagnetic beads. A schematic diagram of the *in vitro* Co-IP experiment under co-transfection conditions was created using the online BioRender website (BioRender.com). The intensity band ratio of Flag (IP) to HA (IP) was quantified using ImageJ software. Densitometric quantification of Flag relative to the HA signal in the co-IP experiments was normalized to the corresponding ratio in the positive control group (vertical lane 2). (**C **and **D**) *In vitro* competitive co-IP analysis of protein–protein interactions under single-transfection conditions. pCAGGS-Flag (10 µg), pCAGGS-sheep-SLAM-Flag (10 µg), pCAGGS-sheep-nectin 4-Flag (10 µg), pCAGGS-virulent PPRV F(V-F)-Flag (10 µg), and pCAGGS-Virulent-PPRV HN (V-HN)-HA (10 µg) were individually transfected into 293T cells in a 100 cm dish. After 24 h post-transfection, cell samples were collected for co-immunoprecipitation analysis. The cell lysates were evenly divided into four aliquots, and additional mouse IgG (negative antibody) and purified HN^1D4-4D9^ (1:25 and 1:100) were added, followed by an equal volume of immunomagnetic beads. The schematic diagram for the *in vitro* Co-IP experiment under single transfection conditions was created using the BioRender website (BioRender.com). The intensity band ratio of Flag (IP) to HA (IP) was quantified using ImageJ software. Densitometric quantification of Flag relative to the HA signal in the co-IP experiments was normalized to the corresponding ratio in the positive control group (vertical lane 2).

Having established that HN^1D4-4D9^ inhibits viral adsorption, we next examined whether this antibody interferes with HN’s interaction with its cellular receptors or the viral fusion protein. Under co-transfection conditions, HN^1D4-4D9^ competitively inhibited the association of HN with SLAM and nectin-4 in a dose-dependent manner compared to negative serum and mock controls (Fig. 6A). This finding was corroborated by an *in vitro* Co-IP assay using a single eukaryotic expression protein, which confirmed that HN^1D4-4D9^ specifically disrupts HN binding to SLAM and nectin-4 but not to the F protein (Fig. 6C). These biochemical assay data provide direct evidence that HN^1D4-4D9^ blocks HN-receptor interactions. In contrast, competitive Co-IP analysis demonstrated that HN^1D4-4D9^ did not disrupt the interaction between HN and F glycoproteins (Fig. 6B and D). These functional interference results indicate that HN^1D4-4D9^ neutralizes PPRV primarily by preventing receptor engagement and viral adsorption, without negatively affecting the biological function of the HN–F complex.

### Determination of the influence of the key epitope sites on the interaction between HN and the viral receptor

As shown in Fig. 3D and Fig. S4, the identified epitope residues (E380, C381, L382, V383, E384, A385, C386, and K387) were localized to an alpha-helical region in the three-dimensional structural simulation of the HN protein. Notably, the epitope recognized by HN^1D4-4D9^ is proximal to, yet distinct from, the identified SLAM receptor-binding interface residues (positions 389, 464, 498, 503, 533, 541, and 543) (Fig. S8A). To further verify the binding stability and dynamic interaction properties of the HN-receptor complex, molecular dynamics simulations were performed on the optimal complexes using the GROMACS software package. Molecular docking simulations suggested that epitope deletion did not significantly affect the interaction between HN and its receptors (SLAM and nectin-4). This conclusion is supported by the predicted binding parameters, including binding modes, interfacial stability, and dynamic behavior, observed for both epitope-deleted and full-length HN complexes (Fig. S8B and C).

**Fig 7:**
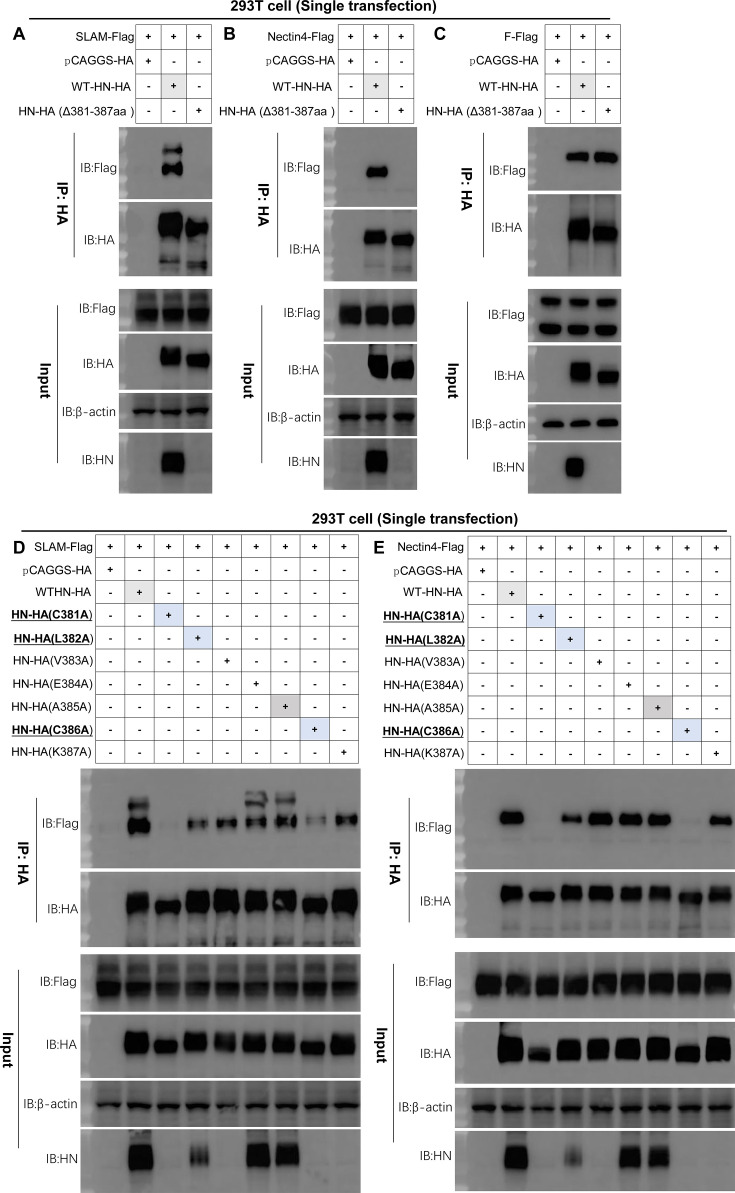
Determination of the effect of key epitope sites on the interaction between HN and the viral receptor. (**A**, **B**, and **C**) *In vitro* competitive Co-IP analysis of HN epitope-deleted mutants with the viral receptor and F glycoprotein. pCAGGS-HA (10 µg), pCAGGS-HN(Δ381–387aa)-HA, pCAGGS-HN-HA, pCAGGS-sheep-SLAM-Flag (10 µg), pCAGGS-sheep-nectin-4-Flag (10 µg), and pCAGGS-F-Flag (10 µg) were individually transfected into 293T cells in a 100 cm dish. After 24 h post-transfection, cell samples were collected for co-immunoprecipitation analysis. (**D** and **E**) *In vitro* competitive co-IP analysis of the interactions between HN epitope mutants and viral receptors. pCAGGS-HA (10 µg), pCAGGS-HN-HA (10 µg), pCAGGS-HN(C381A)-HA (10 µg), pCAGGS-HN(L382A)-HA (10 µg), pCAGGS-HN(V383A)-HA (10 µg), pCAGGS-HN(E384A)-HA (10 µg), pCAGGS-HN(A385A)-HA (10 µg), pCAGGS-HN(C386A)-HA (10 µg), pCAGGS-HN (K387A)-HA (10 µg), pCAGGS-sheep-SLAM-Flag (10 µg), and pCAGGS-sheep-nectin-4-Flag (10 µg) were individually transfected into 293 cells in a 100 cm dish. After 24 h post-transfection, cell samples were collected for co-immunoprecipitation analysis.

To further characterize and confirm the interference of the epitope motif with HN binding to SLAM and nectin-4, we performed an *in vitro* Co-IP assay using a single eukaryotic expression. As shown in Fig. 7, compared with the positive control (lane 2), we unexpectedly observed that the HN epitope-deletion mutant (Δ380–385) failed to interact with either SLAM or nectin-4 (Fig. 7A and B, lane 3), which is inconsistent with the molecular dynamics simulations shown in Fig. S8B and C. Notably, the HN epitope-deletion mutant did not affect its interaction with the viral F glycoprotein (Fig. 7C, lane 3), which is in agreement with the interaction analysis shown in Fig. 6B and D.

**Fig 8:**
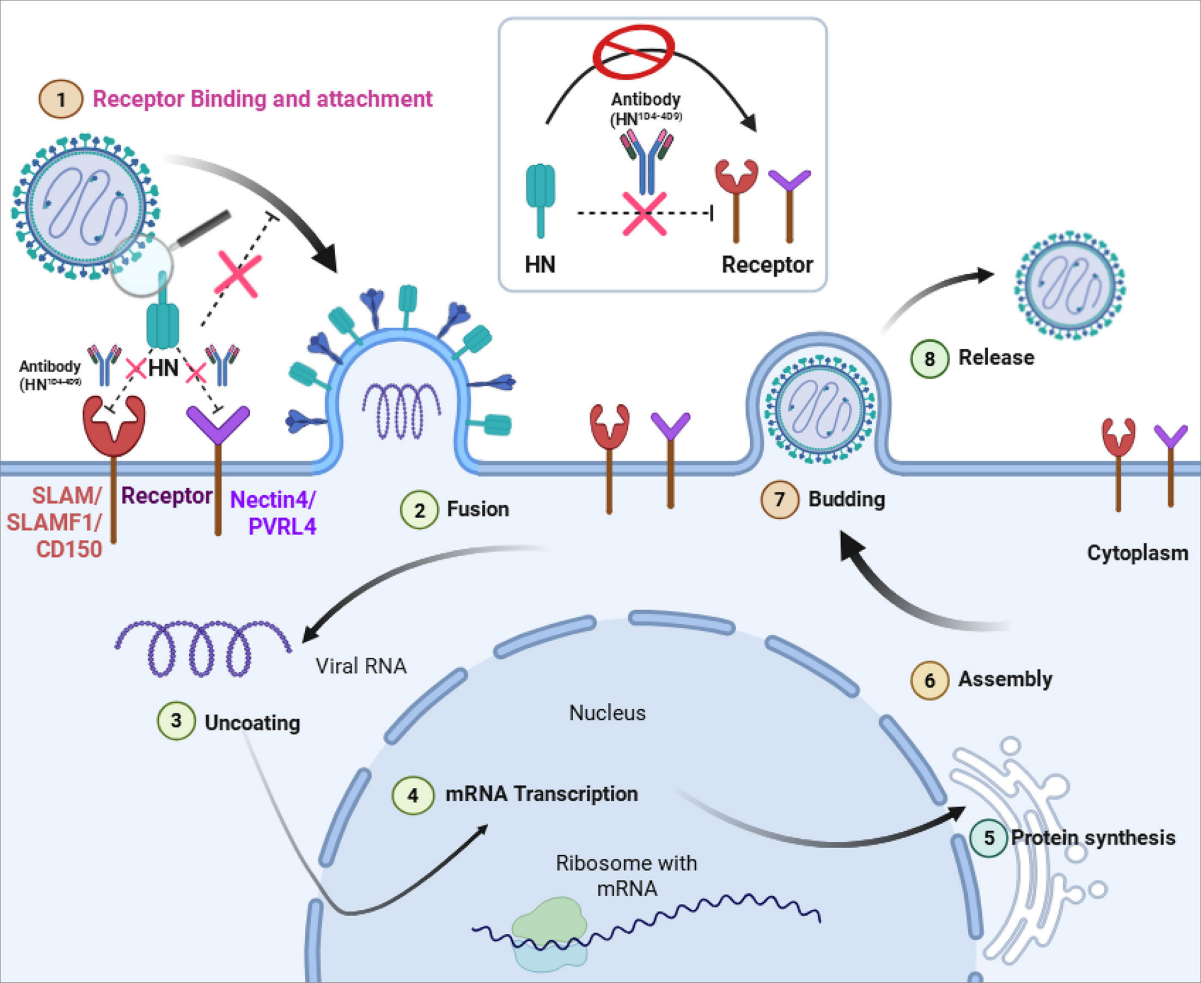
Schematic diagram illustrating the potent neutralizing activity of HN^1D4-4D9^ against PPRV by blocking viral adsorption and receptor engagement. Generally, the HN glycoprotein of PPRV binds to cellular receptors and mediates viral attachment to susceptible host cells by interacting with the SLAM (SLAMF1, also known as CD150) and nectin-4 (PVRL4) receptors (2). Upon attachment, the HN protein triggers the F protein to mediate membrane fusion of the viral envelope with the host cell membrane, allowing the viral genome to enter the host cell (2). In this study, HN^1D4-4D9^ demonstrated potent neutralizing activity against PPRV by blocking viral adsorption and receptor engagement *in vitro*. The schematic diagrams were created using the BioRender website (BioRender.com).

Next, to directly determine the contribution of key epitope residues to the interaction between HN and viral receptors, we performed an *in vitro* Co-IP assay using seven alanine-scanning mutants derived from those shown in Fig. 3A. As shown in Fig. 7D and E, compared with wild-type HN (WT-HN, lane 2) and the synonymous control mutation (A385A, lane 7), Co-IP analysis revealed that residue C381 (lane 3) is essential for HN interaction with both SLAM and nectin-4, as this mutation abolished HN binding to both receptors (SLAM and nectin-4). In contrast, mutations at residues L382 (lane 4) and C386 (lane 8), particularly C386, significantly reduced, but did not eliminate receptor-binding ability, suggesting that these two residues play a contributory but non-essential role in complex recognition. Collectively, these data indicate that three key amino acid residues (C381, L382, and C386) within the HN epitope motif are critical for maintaining functional integrity in their interactions with the SLAM and nectin-4 receptors. These biochemical findings provide direct evidence that the functional binding of HN^1D4-4D9^ to its distinct epitope may induce spatial steric hindrance around the epitope or epitope masking, thereby blocking HN from binding to the SLAM and nectin-4 receptors(Fig. 8).

## DISCUSSION

To elucidate the functional antigenic determinants of the HN glycoprotein and its role in PPRV pathogenesis, we developed a novel mAb, HN^1D4-4D9^. Using this antibody, we systematically mapped and identified a previously unrecognized, highly conserved epitope across multiple PPRV genotypes. Importantly, HN^1D4-4D9^ exhibited potent neutralizing activity *in vitro*. Mechanistic analyses further demonstrated that this antibody inhibits viral infection by blocking viral adsorption and sterically hindering interactions between the HN glycoprotein and the cellular receptors SLAM and nectin-4, rather than interfering with the fusion protein (Fig. 7 and 8).

The identification and characterization of B-cell epitopes are central to understanding antiviral humoral immunity and the rational design of epitope-based vaccines and therapeutics (20, 34, 35). B-cell epitopes are classically classified as either linear, comprising continuous amino acid sequences, or conformational, formed by residues spatially juxtaposed by protein folding (34, 35). While many antibodies typically bind to conformational epitopes on native proteins, which are discontinuous assemblies of residues created by the three-dimensional folding of a protein (34, 35), our epitope mapping data revealed that HN^1D4-4D9^ binds to a single, continuous epitope motif (residues 380–387) that is recognized in both native and denatured states of the PPRV HN. This newly defined neutralizing epitope is located within the alpha-helix of PPRV HN and does not overlap with the two previously reported discontinuous immunodominant amino acid regions associated with PPRV neutralization (36). This observation suggests that the alpha-helix conformation of this region is critical for maintaining the antigenic architecture required for effective recognition by HN^1D4-4D9^. Notably, the ability of HN^1D4-4D9^ to bind this epitope under both native and denaturing conditions further supports the structural stability and functional relevance of this antigenic site. Currently licensed live attenuated vaccine strains, such as Nigeria 75/1 and Sungri 96, belong to genotype II, which is genetically distinct from the now predominant genotype IV field strains circulating in many endemic regions (3). Therefore, the high degree of conservation of this epitope among PPRV genotypes I–IV highlights a critical vulnerability in the viral attachment machinery. Whether viral escape mutations affect viral fitness and antigenicity across different PPRV genotypes warrants further investigation. From evolutionary and translational perspectives, this conserved antigenic site provides a rational target for the development of epitope-based antivirals, next-generation vaccine platforms (e.g., subunit and mRNA vaccines), and sensitive serological assays. In addition, this epitope sequence may serve as a molecular probe for structural and functional studies of the PPRV HN protein, facilitating deeper insights into virus–receptor interactions and mechanisms of viral entry (20, 21, 23, 37).

Antibody-mediated neutralization is a fundamental component of protective humoral immunity against viral infections (35). The development of B-cell–derived neutralizing monoclonal antibodies has proven indispensable for advancing immunotherapies, diagnostics, and vaccine research (21, 23). Clinically, virus-neutralizing mAbs such as nirsevimab and palivizumab have been successfully deployed against RSV (38, 39), whereas MEDI8852 (40), MHAA4595A (41), and VIS410 (33) have shown therapeutic potential against IAV. These precedents underscore the broad translational value of these neutralizing antibodies. In the present study, HN^1D4-4D9^ markedly inhibited PPRV replication and exhibited robust neutralizing activity *in vitro*, consistent with observations in MeV, where neutralizing antibodies predominantly target the H protein (16). Therefore, several avenues for future research are warranted. First, given that PPRV across multiple genotypes maintains a single serotype, HN^1D4-4D9^ could be explored for post-exposure prophylaxis or therapeutic intervention, particularly in high-value animal populations. Second, analogous to oligoclonal nanobody-based antivenoms (42), antibody cocktails combining HN^1D4-4D9^ with antibodies targeting distinct epitopes on HN or F glycoproteins may enhance breadth and potency. Third, HN^1D4-4D9^ may be adapted for diagnostic applications, including PPRV detection and quantification of serum-neutralizing antibody titers (17, 18). Fourth, similar to mAb-1347, which targets the CDV H glycoprotein (43), HN^1D4-4D9^ is a valuable molecular tool for dissecting the sequential steps of PPRV entry into the host cell. A significant limitation of the present study is the lack of *in vivo* validation in the natural host, due to the absence of an established small animal model, the high cost of small ruminants, and biosafety constraints. Future challenge studies in sheep or goats using multiple virulent PPRV strains are essential to confirm the protective efficacy of our *in vitro* findings and to assess their clinical application.

Previous studies have shown that glycoprotein H-specific antibodies are the major contributors to the formation of MeV-induced neutralizing antibodies (16). Neutralizing epitopes on the H protein of MeV can be positioned either proximal or distal to the receptor-binding site (RBS), resulting in distinct mechanisms of action (44). Neutralizing antibodies that bind near or overlap with the RBS typically block receptor engagement, whereas antibodies targeting distal regions may inhibit infection by disrupting H–F interactions (44). Representative examples include mAb C12H5, which targets the RBS on different IAV genotypes (31), and cross-neutralizing antibodies targeting cryptic RBS sites on SARS-CoV-2 variants (27). Another typical example is mAb-1347, which targets the CDV H protein and efficiently ablates syncytia formation without influencing the interaction of the H-SLAM complex (43). Similarly, the structural basis of D25-prefusion RSV F trimer complexes has revealed their potent neutralizing activity against RSV by binding to a quaternary epitope at the trimer apex, thereby locking the fusion glycoprotein in its prefusion state (26). In our study, the viral adsorption assay demonstrated that HN^1D4-4D9^ primarily impaired viral attachment and was ineffective after viral internalization. Competitive Co-IP and functional interference assays further revealed that HN^1D4-4D9^ directly weakens HN interactions with SLAM and nectin-4, rather than the HN–F glycoprotein interaction. Although biochemical assays have demonstrated that three key amino acids (C381, L382, and C386) within the HN epitope motif retain critical binding functionality toward the SLAM and nectin-4 receptors, whether these mutations confer resistance to neutralization by HN^1D4-4D9^ warrants further investigation using recombinant or chimeric PPRV produced via reverse genetics (45). Here, we found that the HN epitope recognized by HN^1D4-4D9^ does not overlap with the critical amino acid residues of PPRV HN that interact with the SLAM receptor but is near several of them, such as R389 (46). Based on the structural characterization of the four-helix bundle in the ectodomains of HN, H, and G among paramyxoviruses (47–49), we propose that the functional binding of HN^1D4-4D9^ to its distinct epitope may cause spatial steric constraints or epitope masking, blocking access to both the adjacent SLAM-binding surface and the nearby nectin-4 binding site on the PPRV HN receptor-binding surface, despite the precise location and sequence of the nectin-4-binding site remaining undefined. Understanding the direct interaction between antibodies and receptors is a key scientific question. Similar to the crystal structures of MeV-H SLAM complexes (50), it remains to be determined whether this epitope directly overlaps the RBS of HN or is allosterically coupled to it. High-resolution direct structural mapping studies, such as cryo-electron microscopy or X-ray crystallography of the HN-HN^1D4-4D9^ complex, will be essential for defining the atomic basis of receptor blockade and fully elucidating the neutralization mechanism of this antibody. Meanwhile, a comparative analysis of effector functions, particularly Fc receptor-mediated activities (e.g., ADCC and ADCP) and complement-dependent cytotoxicity, would help further determine whether the mechanism of action of HN^1D4-4D9^ against PPRV is similar to that of other mAbs, such as 2H5-A14 (24).

### Conclusion

In this study, we successfully generated a novel mAb, designated HN^1D4-4D9^, directed against the HN glycoprotein of PPRV and identified a previously unrecognized, highly conserved linear and conformational epitope across multiple PPRV genotypes. The delineation of this conserved antigenic site not only advances our understanding of HN structure–function relationships but also establishes a well-defined target for the rational development of next-generation vaccines, including subunit and mRNA platforms, as well as epitope-focused antiviral strategies. Functional analyses demonstrated that HN^1D4-4D9^ possesses strong neutralizing activity *in vitro*, primarily by blocking viral adsorption and preventing engagement of the cellular receptors SLAM and nectin-4, rather than by disrupting the HN–F glycoprotein interaction. Collectively, these findings highlight the translational potential of HN^1D4-4D9^ as a candidate for passive immunoprophylaxis, a reagent for improved diagnostic assays, and a precise molecular probe for fundamental studies on PPRV entry mechanisms. Overall, this study provides a conceptual and technical framework that may accelerate efforts toward the effective control and eventual eradication of PPR.

## MATERIALS AND METHODS

### Cells, viruses, plasmids, and reagents

293T and wild-type Vero cell lines were cultured in Dulbecco’s Modified Eagle Medium (DMEM) supplemented with 10% fetal bovine serum (FBS) (ExCell, China). The attenuated PPRV (Nigeria 75/1 strain) was isolated from a commercial vaccine. The green fluorescently labeled PPRV recombinant vaccine strain (PPRV-EGFP), which was rescued from the full-length cDNA clone of the attenuated Nigeria 75/1 vaccine strain, was kindly provided by Dr. Chao Gong (Xinjiang Agricultural University). The plasmids, including pCAGGS, pET28a, and lentiviral vectors (pLv-puro-sheep-derived SLAM, pLv-Hygro-sheep-derived nectin-4, pMD2.G, and psPAX2), were kindly provided by Prof. Zengqi Yang (Northwest A&F University). Full-length HN and F eukaryotic expression plasmids (pCAGGS-V-HN-HA and pCAGGS-V-F-Flag) derived from a virulent PPRV strain were constructed in our laboratory. All mutant and deletion variants of the PPRV HN gene were constructed by homologous recombination. An HA tag was fused to the C-terminus to avoid disrupting the native N-terminal signal peptide and ectodomain structure, thereby facilitating protein detection and maintaining protein function.

Primary antibodies, including mouse anti-PPRV NP, anti-sheep SLAM, and anti-sheep nectin-4 polyclonal antibodies, were kindly provided by Dr. Chao Gong (Xinjiang Agricultural University) and Prof. Zengqi Yang (Northwest A&F University). Anti-β-actin antibody was purchased from Nulen Biotechnology (Shanghai, China). Anti-Flag and anti-HA antibodies were obtained from Abmart Biotechnology. Secondary antibodies, including horseradish peroxidase-conjugated (HRP) goat anti-rabbit or goat anti-mouse IgG antibodies, were purchased from CWBio (Jiangsu, China). Goat anti-mouse and goat anti-rabbit Alexa Fluor secondary antibodies, such as Alexa Fluor 488 and 594-conjugated antibodies (Molecular Probes), used for indirect immunofluorescence, were purchased from Thermo Fisher Scientific (Waltham, MA, USA). The 2× Phanta Flash Master Mix and Phanta Max Super-Fidelity DNA Polymerase used for PCR were purchased from Vazyme Biotechnology (Nanjing, China). Hieff qPCR SYBR Green Master Mix (No Rox) for real-time quantitative PCR was obtained from YEASEN Biotechnology (Shanghai, China). The Clone Express Ultra One Step Cloning Kit V2, used for plasmid construction, was purchased from Vazyme Biotechnology (Nanjing, China).

### Construction of Vero cell lines that stably express sheep-derived SLAM and nectin-4 receptors

The Vero cell line stably expressing sheep-derived SLAM and nectin-4 (Vero-SN) was constructed using a lentiviral packaging system. Briefly, the optimal concentrations of antibiotics (puromycin and blasticidin) required to kill 100% of wild-type Vero cells were first determined. High-quality, endotoxin-free plasmid DNA was prepared, including pLv-puro-sheep-derived SLAM, pLv-Hygro-sheep-derived nectin-4, pMD2.G, and psPAX2. To generate the Vero-puro-SLAM cell line, pMD2.G, psPAX2, and pLv-puro-sheep-SLAM were transfected into 293T cells at approximately 80%–90% confluency in six-well plates for lentiviral packaging. A total of 2 μg of DNA was transfected at a ratio of 2:1:2 using FuGENE HD (Promega, Madison, WI, USA). After 24 h of lentiviral transduction, the medium was replaced with puromycin (4 μg/mL), and the first round of drug screening was performed until all non-transduced cells died. Approximately 20 well-isolated colonies (Vero-SLAM) were picked into a 96-well plate and validated by Western blot or indirect immunofluorescence assay. Finally, a Vero-SLAM-nectin-4 cell line was generated from the Vero-SLAM cell line. Vero-SLAM-nectin4 cells were generated using the same lentiviral packaging and transduction procedures, followed by hygromycin (250 μg/mL) selection. Drug-resistant colonies (Vero-SLAM-Nectin4, Vero-SN) were selected, expanded, and validated as previously described.

### Prokaryotic expression and generation of monoclonal antibody (mAb) against PPRV HN

Considering that the PPRV HN protein is a type II envelope glycoprotein, bioinformatics analyses were performed to identify dominant antigenic epitope regions, signal peptide, hydrophobicity, and transmembrane regions. A truncated version of the HN protein (223–551 aa) was selected for prokaryotic expression and protein induction under codon optimization. The pET28a-PPRV-HN plasmid was constructed using homologous recombination. After protein expression and purification, mAbs against the HN protein of PPRV were produced by immunizing BALB/c mice with the purified recombinant PPRV HN protein. Mice received an initial subcutaneous injection of 75 µg antigen emulsified in complete Freund’s adjuvant, followed by intraperitoneal boosts with the same amount of antigen in incomplete Freund’s adjuvant at 14-day intervals. Three days after the final immunization, splenocytes from the highest responders were fused with SP2/0 myeloma cells using polyethylene glycol. Hybridomas were selected in HAT medium and cloned by limiting dilution. Supernatants were screened for HN-specific antibodies using ELISA and Western blot. The hybridoma clone 1D4-4D9 was selected for ascitic production in mice. The mAb class and subclass (IgG1, IgG2a, IgG2b, IgG3, IgM, IgA, kappa, lambda) were determined using a mouse mAb isotype/subclass ELISA Kit (6101, Bioablab, Luoyang, China). Finally, HN^1D4-4D9^ was purified using Nab Protein A/G Spin Columns (89958, Thermo Fisher) for subsequent experiments.

### Indirect immunofluorescence assay analysis

Transfected wild-type Vero or Vero-SN cells were fixed with 4% paraformaldehyde, permeabilized with 0.5% Triton X-100, and blocked with 5% BSA. The cells were sequentially incubated with primary antibodies, followed by fluorescent secondary antibodies (Alexa Fluor 488 or 594). Nuclei were counterstained with DAPI, and the coverslips were mounted on slides for imaging using a ZEISS LSM880 confocal microscope.

### Western blot and dot blot analyses

Cells (293T, Vero, or Vero-SN) were lysed, and the clarified supernatants were denatured in 1× SDS buffer. Proteins were separated by SDS-PAGE, transferred to nitrocellulose membranes, and detected using specific primary and horseradish peroxidase-conjugated secondary antibodies. Finally, the antibody–antigen complex was exposed with a chemiluminescence reagent solution kit (US EVERBRIGHT, Suzhou, China) using a multi-chemiluminescence imaging system (GE, Amersham Imager 600 or Bio-Rad, ChemiDoc MP).

For dot blot analysis, the synthetic peptides were directly spotted onto a nitrocellulose membrane and serially diluted. The membrane was then processed according to the Western blot protocol, including blocking, incubation with an anti-HN^1D4-4D9^ primary antibody, and an HRP-conjugated secondary antibody, followed by chemiluminescent detection. Both assays were imaged using a multi-chemiluminescence imaging system.

### Co-immunoprecipitation analysis

For competitive Co-IP analysis under co-transfection conditions, two plasmids, including pCAGGS, pCAGGS-virulent PPRV HN (V-HN)-HA, pCAGGS-sheep SLAM-Flag, pCAGGS-sheep nectin-4-Flag, or pCAGGS-virulent PPRV F (V-F)-HA, were co-transfected into 293T cells. After 24 h post-transfection, lysates from cells overexpressing these two proteins (HN and its interactors) were harvested and lysed in ice-cold lysis buffer for 30 min on ice with occasional vortexing. The lysate was clarified by centrifugation at 12,000 × *g* for 15 min at 4°C, and the supernatant was transferred to a new pre-chilled tube. An additional negative control antibody (IgG) or purified HN^1D4-4D9^ (1:25 and 1:100) as a competitor was added before supplementation with an equal volume of immunomagnetic beads (HY-K0201, Med Chem Express, USA). After overnight incubation at 4°C, the immunomagnetic beads were washed three times on a magnetic rack. After the final wash, 45 µL of 2× SDS PAGE loading buffer was added directly to the beads, which were then heated at 100°C for 10 min to denature the proteins and release the complex samples. The samples were then collected using a magnetic rack and processed according to the Western blot protocol.

For *in vitro* Co-IP assays under single-transfection conditions, 293T cells in a 100 cm dish were individually transfected with pCAGGS, pCAGGS-V-HN-HA, pCAGGS-HN mutant-HA, pCAGGS-V-F-HA, pCAGGS-sheep-derived SLAM-Flag, or pCAGGS-sheep-derived nectin-4-Flag. After 24 h post-transfection, the transfected 293T cells were harvested, lysed, and clarified by centrifugation at 12,000 rpm for 15 min at 4°C to obtain eukaryotic proteins (HN, SLAM, nectin-4, and F). The resulting cell lysates were divided equally into four aliquots and incubated with either control mouse IgG or the purified HN^1D4-4D9^ antibody at dilutions of 1:25 or 1:100, followed by the addition of an equal volume of immunomagnetic beads. After antibody–protein complex formation, the individually expressed proteins were combined and subjected to *in vitro* Co-IP analysis.

### Flow cytometry analyses

Vero-SN cells were grown to 90% confluence in advance after HN^1D4-4D9^ pre-treatment, mixture-treatment, or post-treatment prior to PPRV-EGFP infection (TCID_50_ = 10^4.614^) at an MOI of 1. After 48 h post-infection, the Vero-SN cells were digested with 0.05% trypsin-EDTA at 37°C. The collected cells were collected into 1.5 mL tubes and centrifuged at 2,500 rpm for 5 min at 4 °C. The cell clumps were washed once with PBS supplemented with 2.5% BSA. Lastly, after washing twice with PBS supplemented with 2% BSA, the results of flow cytometry from 10,000 cells were captured using CytoFLEX software (Beckman Coulter, Brea, CA, USA) and analyzed by FlowJo Version 10 software (TreeStar, Ashland, OR, USA).

### Quantitative polymerase chain reaction

For qPCR analysis, Vero-SN cells were cultured in a 12-well plate in advance. After HN^1D4-4D9^ pre-, post-, or mixture-treatment, the Vero-SN cells were infected with PPRV (Nigeria 75/1 strain) at an MOI of 1. Total viral RNA was extracted, reverse-transcribed, and quantified by Hieff qPCR SYBR Green Master Mix. Based on the PPR diagnostic techniques, the national standard of the People’s Republic of China (GB/T 27982-2011), the primer sequences targeting the PPRV NP for the real-time PCR test were as follows: PPRV8a Forward, 5′-CACAGCAGAGGAAGCCAAACT-3′. PPRV9b Reverse, 5′-TGTTTTGTGCTGGAGGAAGGA-3′. Based on the concentration of the standard plasmids PPRV NP, a 10-fold dilution series of the recombinant plasmid was used as the target DNA to generate the standard curve. The concentrations of the standard plasmids were converted to copy numbers to optimize the reaction conditions. A qPCR test was performed, and a standard curve for the recombinant plasmid was generated using the formula. The linear equation for PPRV NP real-time PCR was *Y* = −3.3949*X* + 39.59, with an *R*^2^ value of 0.9982. Melt curve analysis was performed to verify the specificity of the amplified products. The reaction was performed according to the manufacturer’s protocol using a Bio-Rad CFX96 Real-Time PCR System (Applied Biosystems, Foster City, CA, USA). A standard curve was constructed for the subsequent absolute quantification of PPRV genomic copies.

### Indirect enzyme-linked immunosorbent assay analysis

An indirect ELISA was performed to evaluate the binding of the purified HN protein and synthetic polypeptides to HN^1D4-4D9^. Briefly, a 96-well plate was coated with serial dilutions of the purified HN protein (range: 0.0703125–9 µg/mL), single-epitope peptides (CLVEACK) (range: 0.00976562–1.25 µg/mL), and tandem epitope peptides (CLVEACKGGGSCLVEACK) (range: 0.00000298–0.1398 µg/mL) in coating buffer overnight at 4°C. After washing, the plate was blocked for 1 h at room temperature. The plate was then incubated sequentially with the primary antibody (HN^1D4-4D9^, 1:20,000) and an HRP-conjugated secondary antibody (1:5,000), each for 1 h at 37°C, with washing steps in between. The reaction was terminated with a stop solution, and the absorbance was measured using a Varioskan LUX multimode microplate reader (Thermo Fisher Scientific).

### Surface plasmon resonance assay

The binding affinity of HN^1D4-4D9^ was analyzed using SPR with a Biacore 8 K instrument (Cytia). The antibodies were diluted (30 µg/mL) and immobilized on a CM5 sensor chip (29149603, Cytia) via amine coupling. The purified HN protein (range: 31.25–500 nM), the synthesized single-epitope polypeptide (CLVEACK) (range: 390.625–12,500 nM), and the tandem epitope peptides (CLVEACKGGGSCLVEACK) (range: 195.3125–6,250 nM) were serially diluted in running buffer (1× HBS EP) and injected over the chip surface. Binding was measured at a flow rate of 30 µL/min with a 60-second contact and dissociation phase with a 60-second contact. The surface was then regenerated with 100 mM glycine (pH = 3.0). Sensorgrams were reference-subtracted, and binding kinetics were analyzed using Biacore 8K evaluation software, where the apparent equilibrium dissociation constants (*K*_D_) were calculated using a kinetic-fitting affinity model.

### Viral neutralization assay

To evaluate the neutralizing activity of HN^1D4-4D9^, monolayers of Vero-SN cells at 90%–100% confluence were seeded in a 96-well plate 1 day prior to viral infection. After HN^1D4-4D9^ pre-, post-, or mixture treatment for 1 h prior to viral infection, the cells were infected with extracellular mature virions of the Nigeria 75/1 vaccine strain (TCID_50_ = 10^4.806^) at an MOI of 1. After treatment under experimental conditions, the total intracellular amount of viral proteins was quantified by harvesting mock- or PPRV-infected cells, washing them extensively to remove residual extracellular virus, and subjecting the cells to freeze–thaw lysis to release intracellular viral particles. The clarified lysate was subjected to WB analysis. To quantify extracellular infectious viruses, the collected supernatant was centrifuged at 5,000 rpm for 10 min to remove cellular debris, serially diluted, and inoculated onto Vero-SN cell monolayers. After 72 h of viral incubation, cytopathic effects (CPE) were examined microscopically. The TCID₅₀ titer was calculated using the Reed-Muench or Karber method, based on the highest dilution that induced CPE in 50% of the cell culture wells.

For immunofluorescence observation, Vero-SN cells underwent HN^1D4-4D9^ pre-, post-, or mixture-treatment 1 h prior to infection with EGFP-labeled recombinant Nigeria 75/1 vaccine strain (PPRV-EGFP, TCID_50_ = 10^4.614^) at an MOI of 1. After treatment, immunofluorescence intensity and syncytial formation were directly monitored. Representative images were captured using the EVOS M5000 imaging system.

### Bioinformatics analysis

For amino acid and nucleotide sequences, sequence alignments were performed using the CLUSTAL W implemented in DNASTAR (https://www.dnastar.com/software/), Snap Gene software (https://www.snapgene.com/), Jalview (https://www.jalview.com/), and multiple sequence alignment-CLUSTAL W (https://www.genome.jp/tools-bin/clustalw). Conserved residues and positional amino acid variability were visualized using WebLogo 3 (https://weblogo.threeplusone.com/) and multiple MUSCLE (http://www.drive5.com/muscle) (51). The B-cell epitope prediction was performed using the online IEDB analysis resource website (http://tools.iedb.org/bcell/). For epitope prediction and structural simulation, the nucleotide sequence of PPRV HN was translated into the corresponding amino acid sequence using DNASTAR software and exported in the FASTA format. The amino acid sequence was subsequently submitted to the SWISS-MODEL server (https://swissmodel.expasy.org/) and the AlphaFold protein structure database (https://alphafold.com). The predicted structures were downloaded and saved in the Protein Data Bank (PDB) format. Subsequently, structural visualization and refinement were performed using the open-source PyMOL 2.5 software (https://pymol.org/2/). Molecular docking simulations were performed using PyMOL 2.5 software (https://pymol.org/2/) for visualization and GROMACS (https://www.gromacs.org/) for molecular dynamics-based docking and interaction analyses.

### Statistical information analysis

Statistical analyses were performed using GraphPad Prism software (Version 8.4.3.686). Data from a minimum of three independent experiments are presented as mean ± standard error of the mean (SEM). For comparisons of three or more groups, statistical significance was assessed using a one-way ANOVA. For comparisons between two groups, a two-tailed Student’s t-test was used. Significance levels are denoted as follows: ns (not significant) for *P* > 0.05, * for *P* < 0.05, ** for *P* < 0.01, and *** for *P* < 0.001.

## Data Availability

The authors confirm that the data supporting the findings of this study are included in this article or its supplemental material. All data sets generated and/or analyzed during the current study are available from the corresponding authors, Dr. Shanhui Ren or Dr. Jinxin Xie, upon reasonable request.

## SUPPLEMENTAL MATERIAL

Fig. S1 jvi.00787-26-s0001.pdf Preparation of the monoclonal antibody (mAb) targeting the PPRV HN protein.

Fig. S2 jvi.00787-26-s0002.pdf Epitope mapping of the native state of the PPRV HN protein recognized by HN^1D4-4D9^.

Fig. S3 jvi.00787-26-s0003.pdf Epitope mapping of the denatured PPRV HN protein recognized by HN^1D4-4D9^.

Fig. S4 jvi.00787-26-s0004.pdf Spatial structural prediction and simulation of HN proteins of virulent and attenuated PPRV strains.

Fig. S5 jvi.00787-26-s0005.pdf Establishment of a Vero cell line stably expressing SLAM and nectin-4 receptors.

Fig. S6 jvi.00787-26-s0006.pdf Comparative analysis of PPRV infectivity in wild-type Vero and Vero-SN cells.

Fig. S7 jvi.00787-26-s0007.pdf Observation of potent neutralizing activity of HN^1D4-4D9^ against PPRV in the Vero-SN cell model.

Fig. S8 jvi.00787-26-s0008.pdf Molecular docking and dynamic simulations of the PPRV HN protein with cellular receptors.

Supplemental legends jvi.00787-26-s0009.docx Fig. S1-S8 and Tables S1-S2.

ASM does not own the copyrights to Supplemental Material that may be linked to, or accessed through, an article. The authors have granted ASM a non-exclusive, world-wide license to publish the Supplemental Material files. Please contact the corresponding author directly for reuse.
