# Supplementary figures and images for: A novel monoclonal antibody targeting the hemagglutinin–neuraminidase of peste des petits ruminants virus maintains neutralizing activity by blocking viral adsorption and receptor interaction
Source: J Virol. 2026 Jun 26;100(7):e00787-26. doi: 10.1128/jvi.00787-26 (PMC13386946; doi:10.1128/jvi.00787-26)

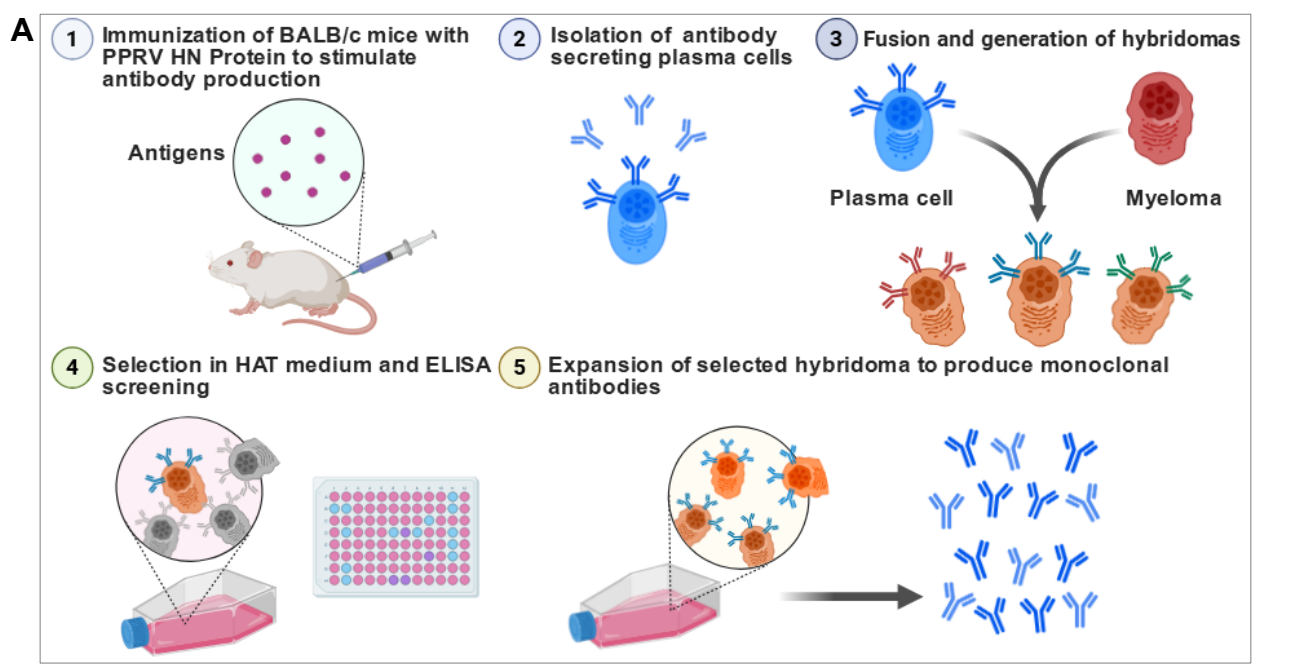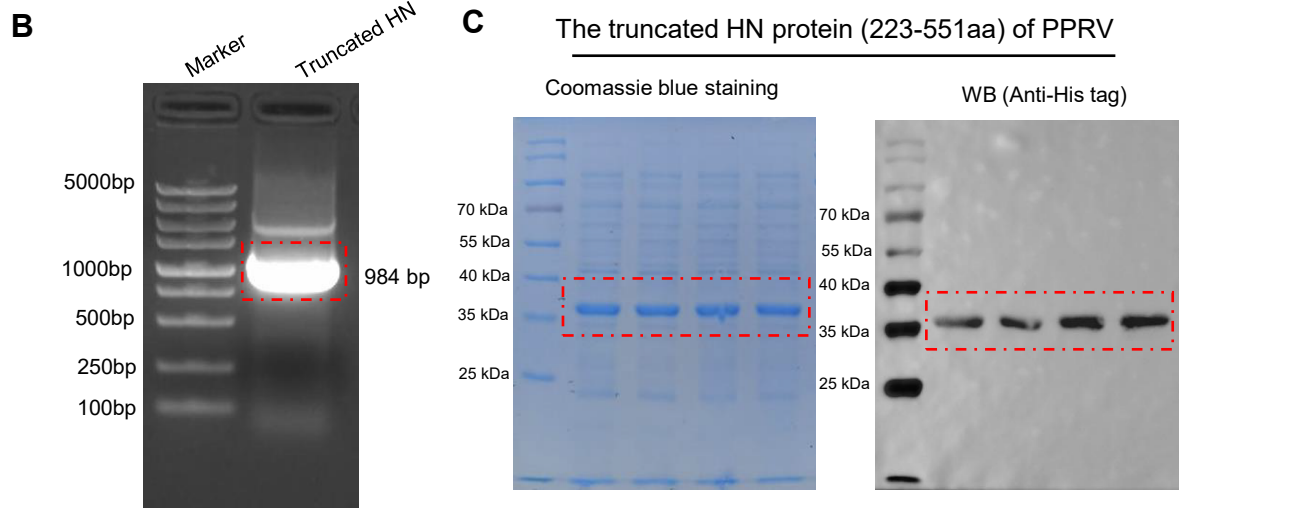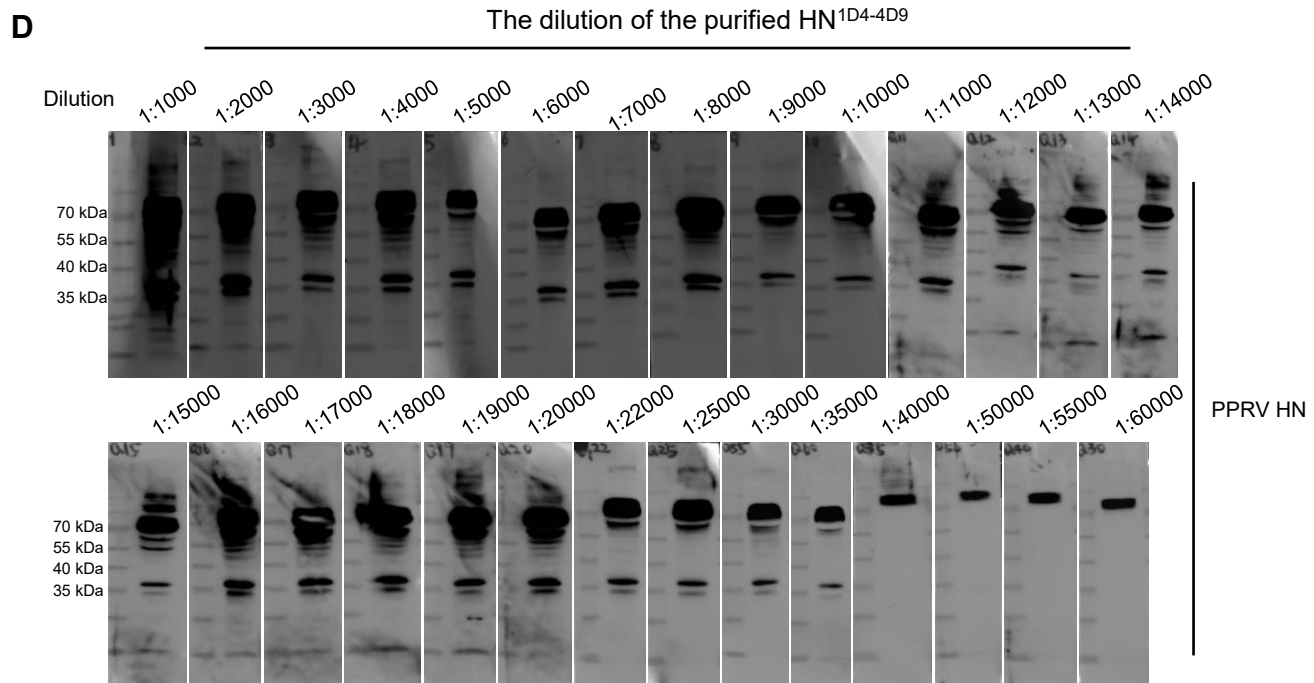

Supplement: Fig. S1 — Preparation of the monoclonal antibody (mAb) targeting the PPRV HN protein. [file jvi.00787-26-s0001.pdf]

# Epitope mapping of the native state of PPRV HN protein recognized by HN<sup>1D4-4D9</sup>

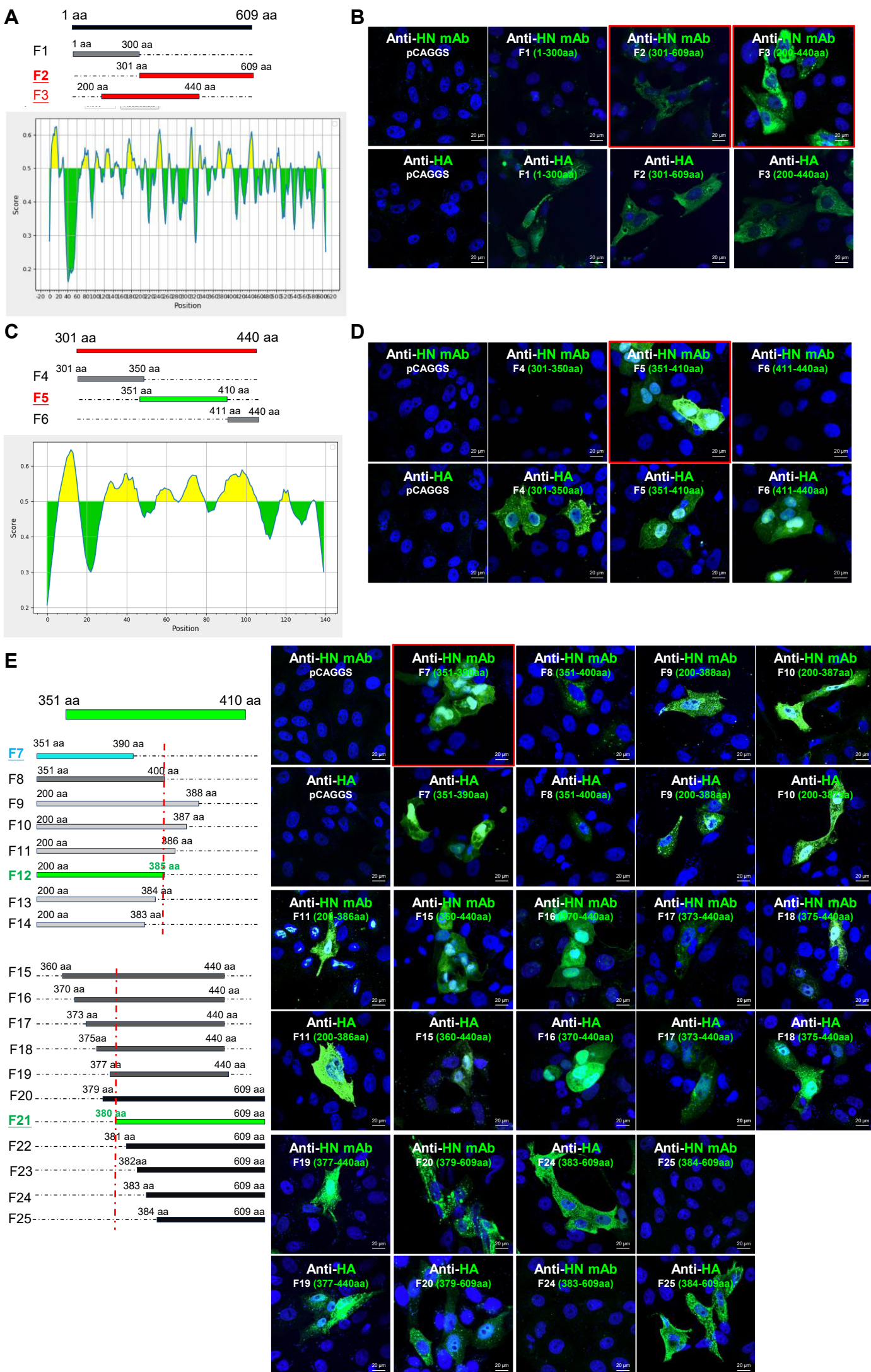

Supplement: Fig. S2 — Epitope mapping of the native state of the PPRV HN protein recognized by HN1D4-4D9. [file jvi.00787-26-s0002.pdf]

# Epitope mapping of the denatured state of PPRV HN protein recognized by HN<sup>1D4-4D9</sup>

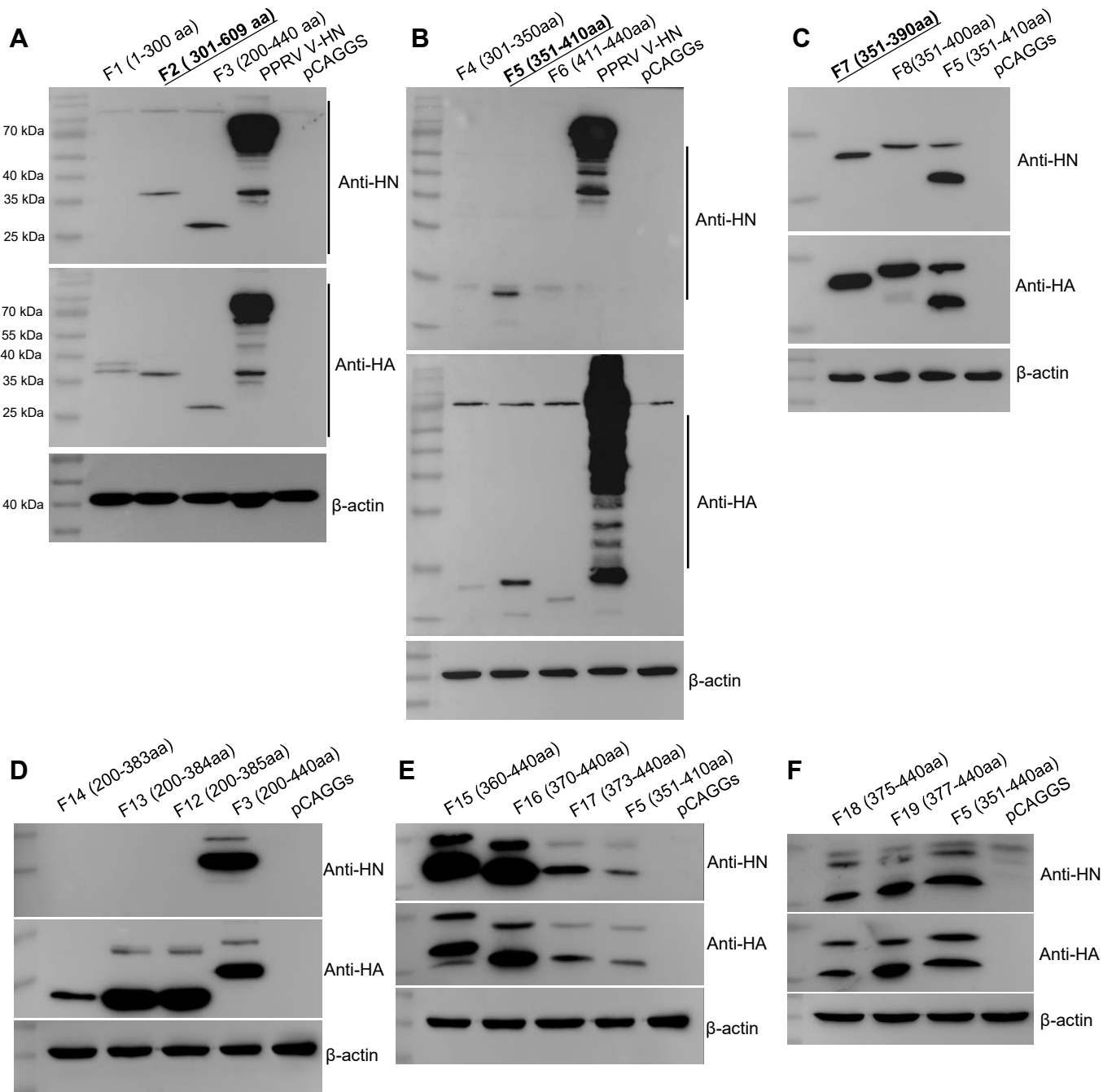

Supplement: Fig. S3 — Epitope mapping of the denatured PPRV HN protein recognized by HN1D4-4D9. [file jvi.00787-26-s0003.pdf]

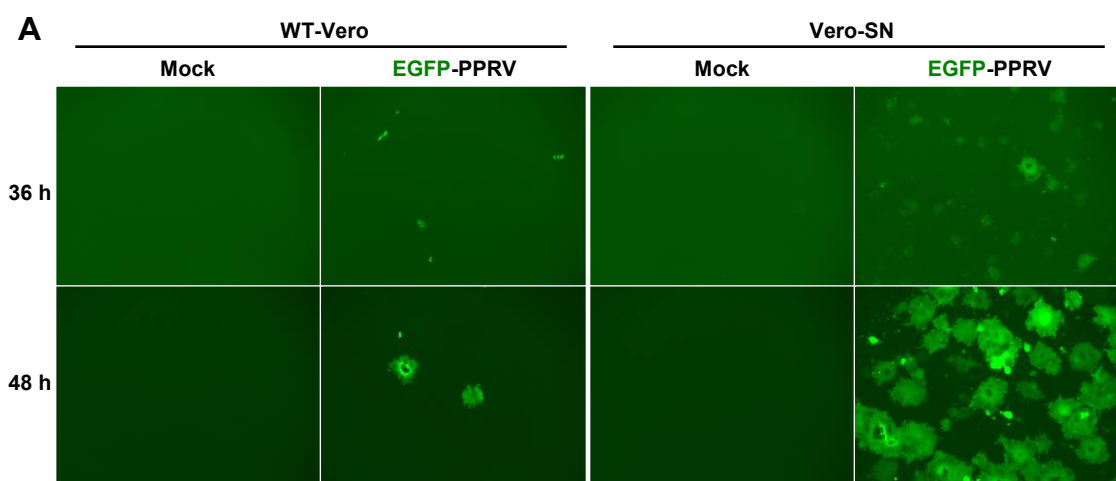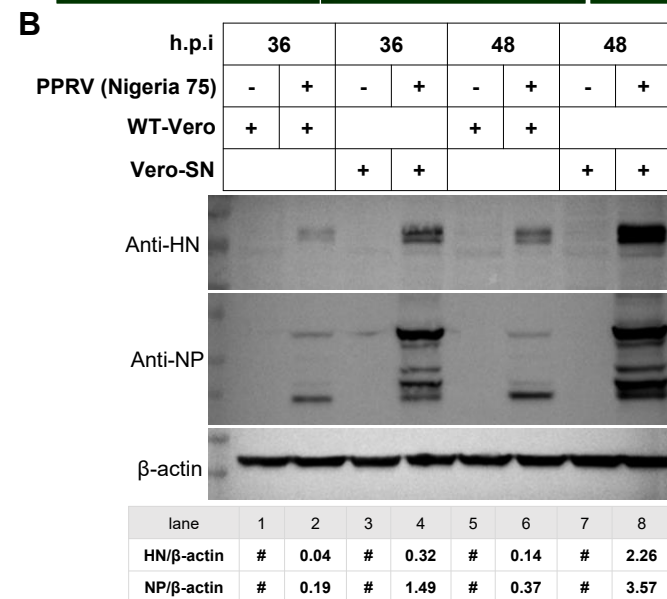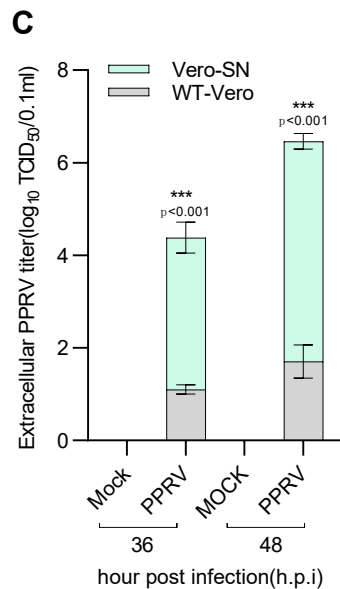

Supplement: Fig. S6 — Comparative analysis of PPRV infectivity in wild-type Vero and Vero-SN cells. [file jvi.00787-26-s0006.pdf]

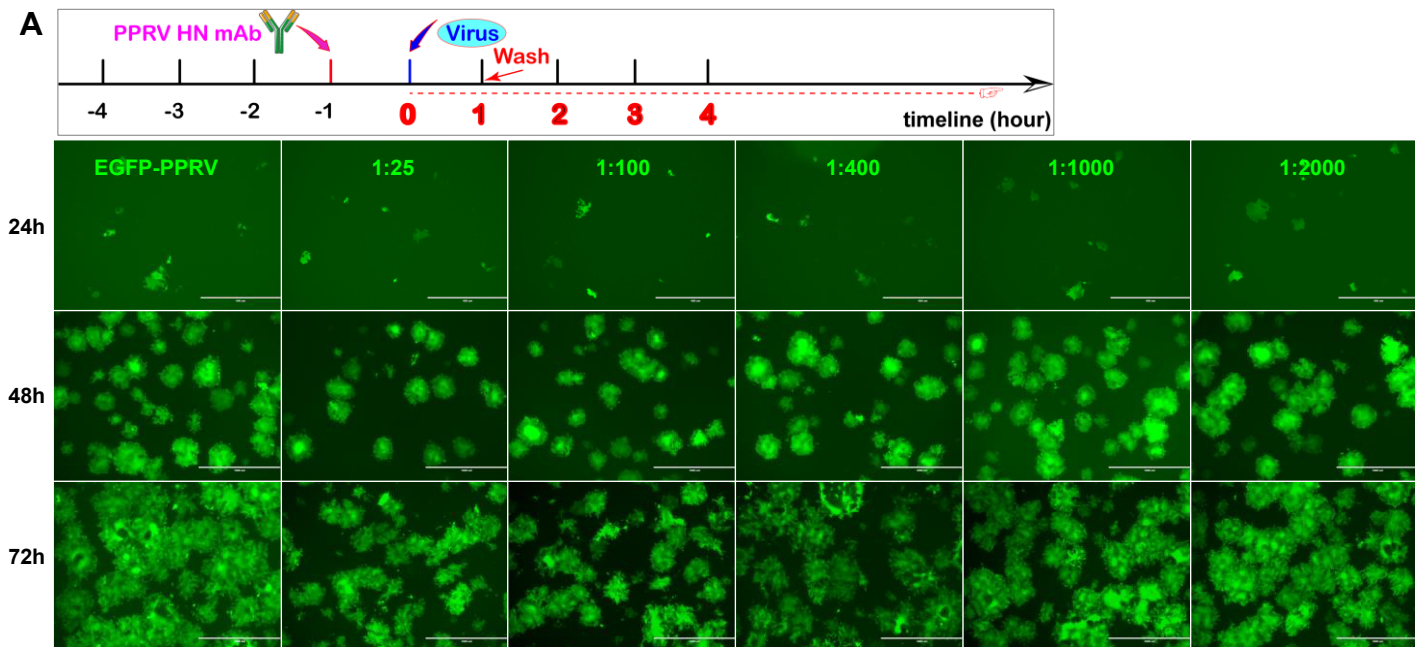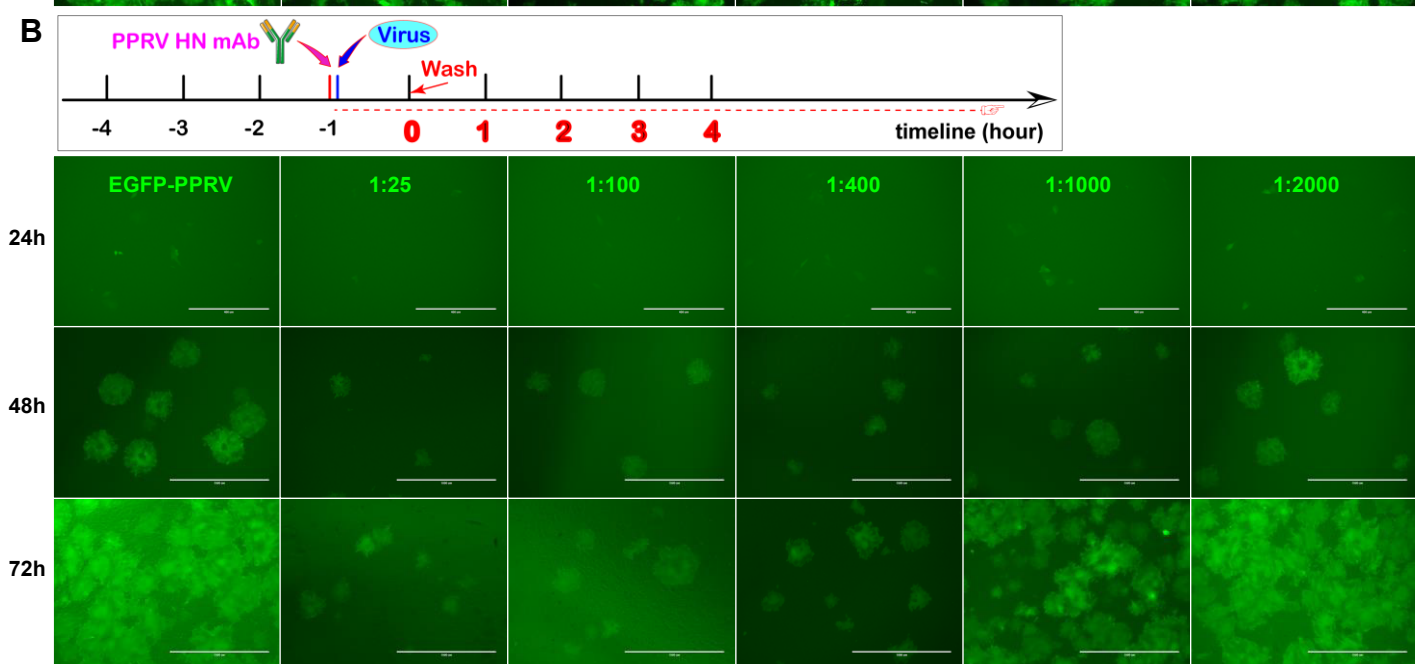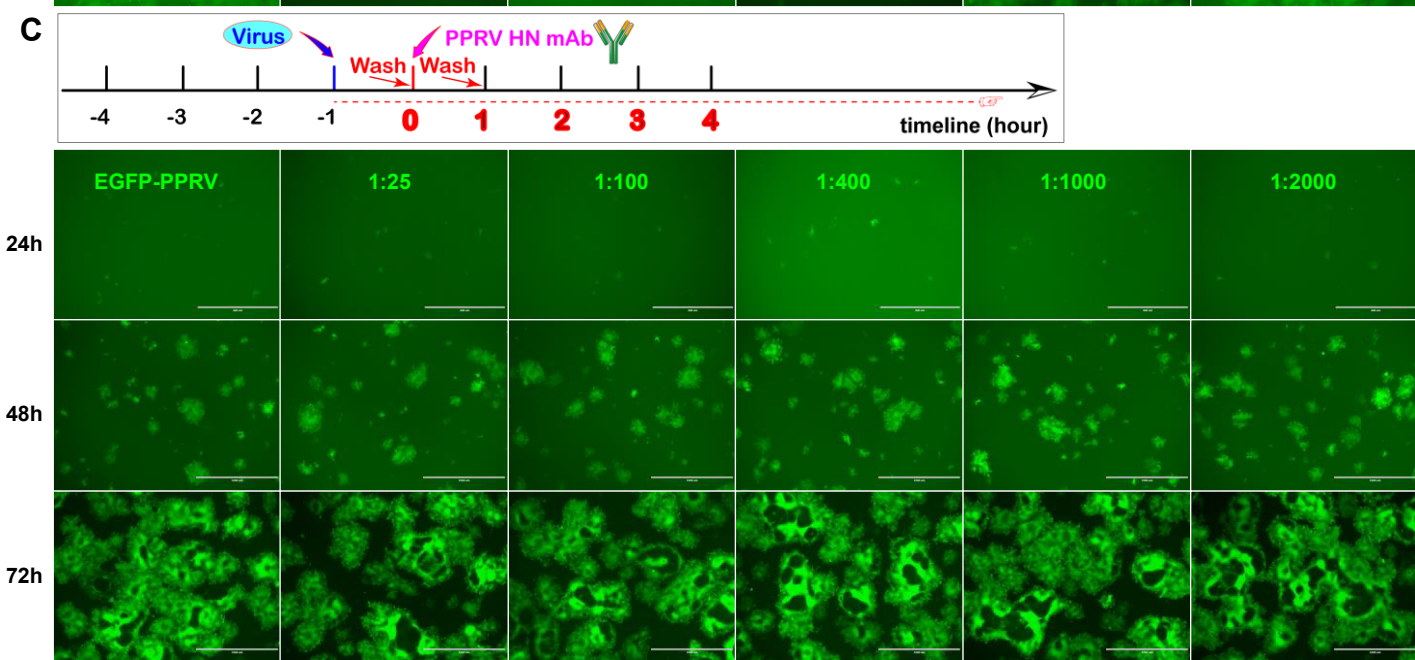

Supplement: Fig. S7 — Observation of potent neutralizing activity of HN1D4-4D9 against PPRV in the Vero-SN cell model. [file jvi.00787-26-s0007.pdf]

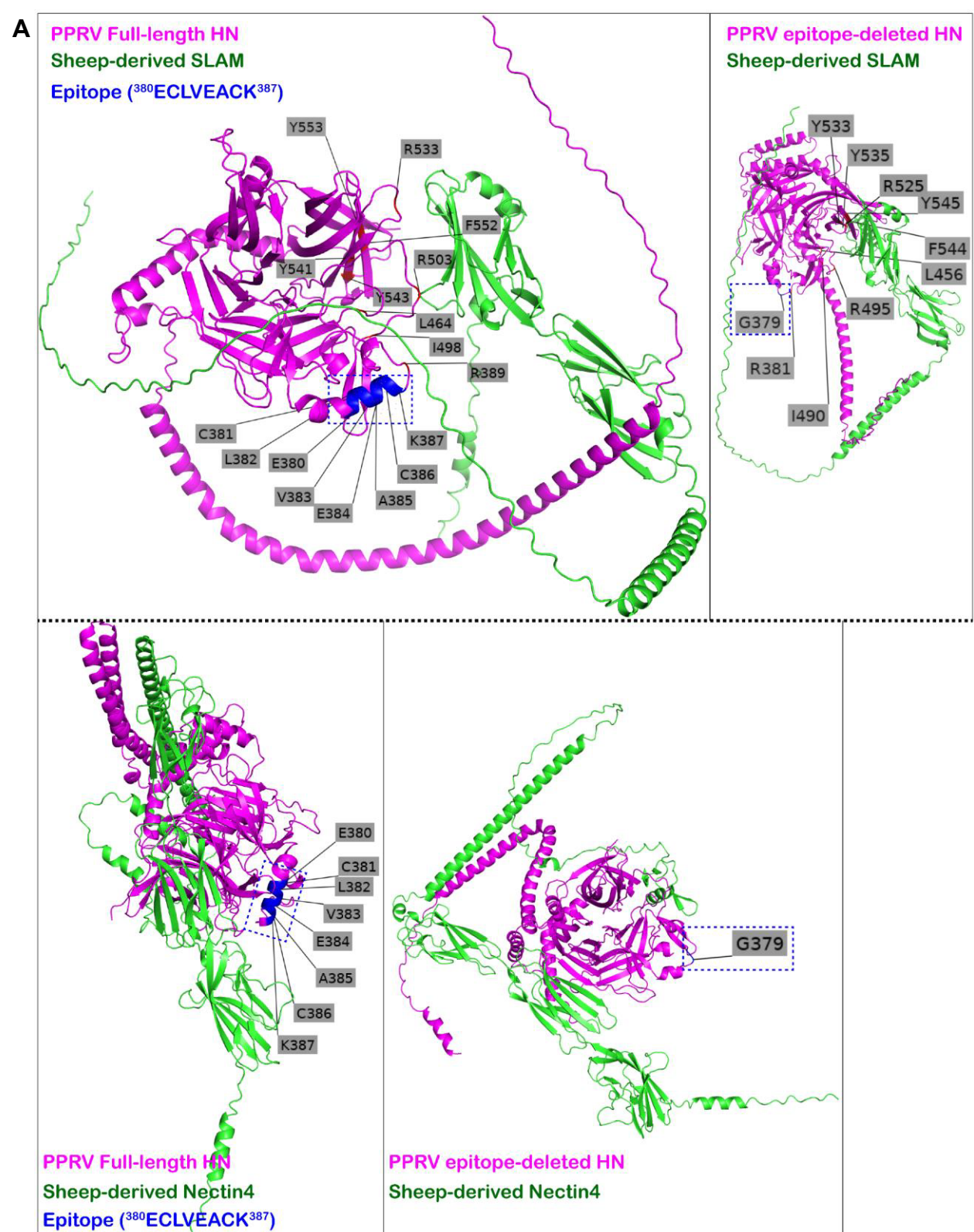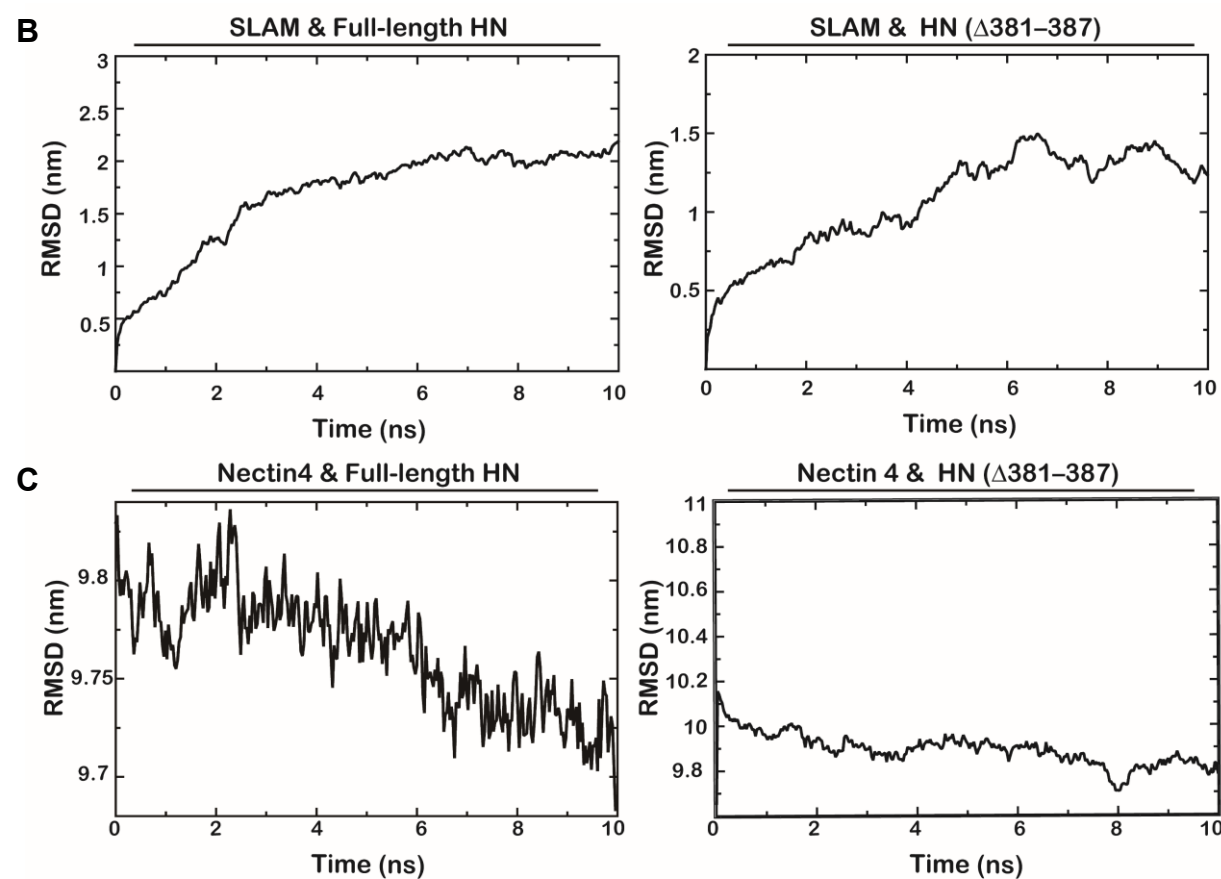

Supplement: Fig. S8 — Molecular docking and dynamic simulations of the PPRV HN protein with cellular receptors. [file jvi.00787-26-s0008.pdf]
