# Supplementary material for: A novel monoclonal antibody targeting the hemagglutinin–neuraminidase of peste des petits ruminants virus maintains neutralizing activity by blocking viral adsorption and receptor interaction
Source: J Virol. 2026 Jun 26;100(7):e00787-26. doi: 10.1128/jvi.00787-26 (PMC13386946; doi:10.1128/jvi.00787-26)
Supplement: Fig. S4 — Spatial structural prediction and simulation of HN proteins of virulent and attenuated PPRV strains. [file jvi.00787-26-s0004.pdf]

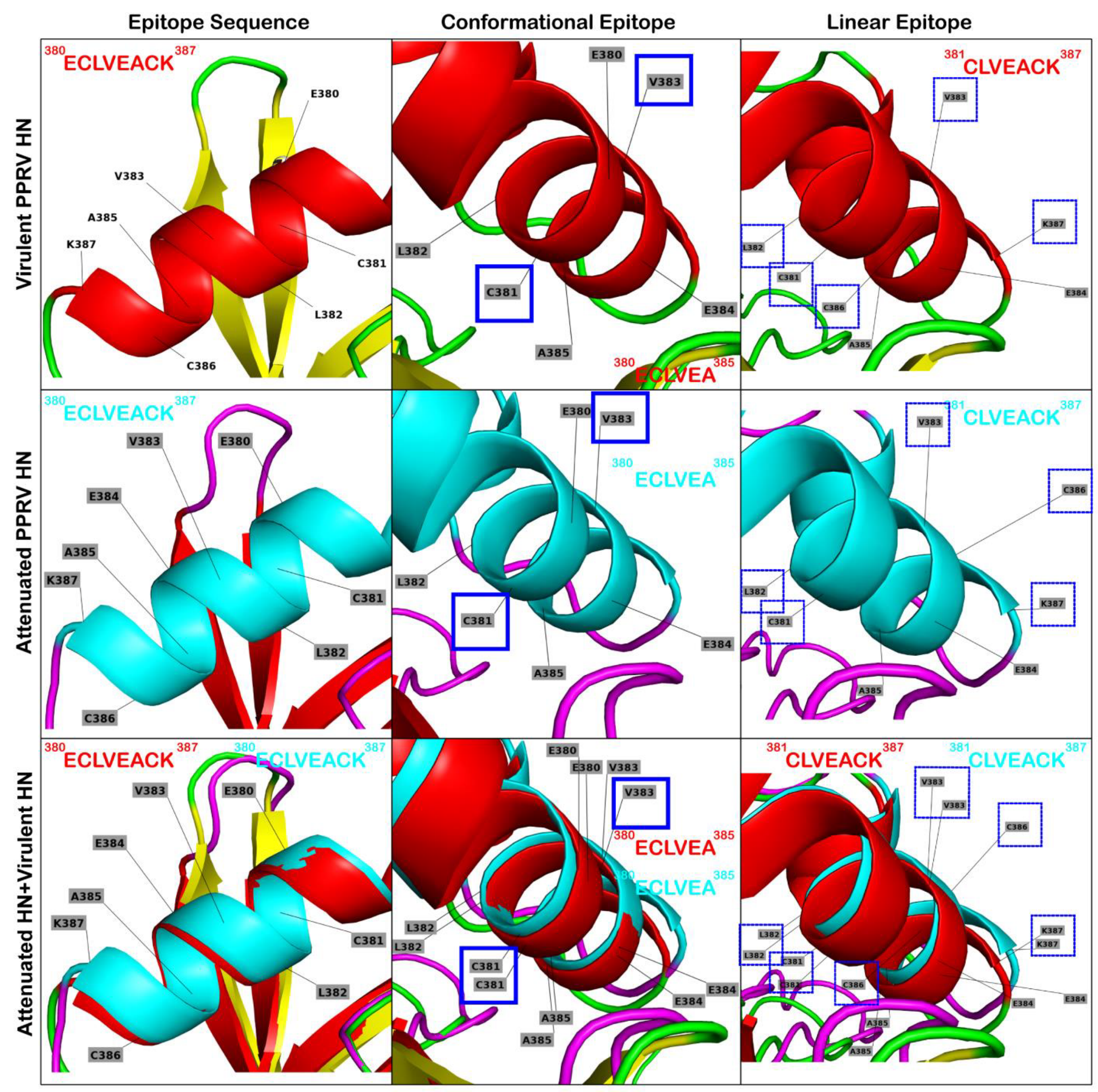
Epitope Sequence
Conformational Epitope
Linear Epitope
Virulent PPRV HN
Attenuated PPRV HN
Attenuated HN+Virulent HN
380 ECLVEACK 387
381 CLVEACK 387
380 ECLVEA 385
E380
C381
L382
V383
E384
A385
C386
K387
