# Supplementary material for: A novel monoclonal antibody targeting the hemagglutinin–neuraminidase of peste des petits ruminants virus maintains neutralizing activity by blocking viral adsorption and receptor interaction
Source: J Virol. 2026 Jun 26;100(7):e00787-26. doi: 10.1128/jvi.00787-26 (PMC13386946; doi:10.1128/jvi.00787-26)
Supplement: Fig. S5 — Establishment of a Vero cell line stably expressing SLAM and nectin-4 receptors. [file jvi.00787-26-s0005.pdf]

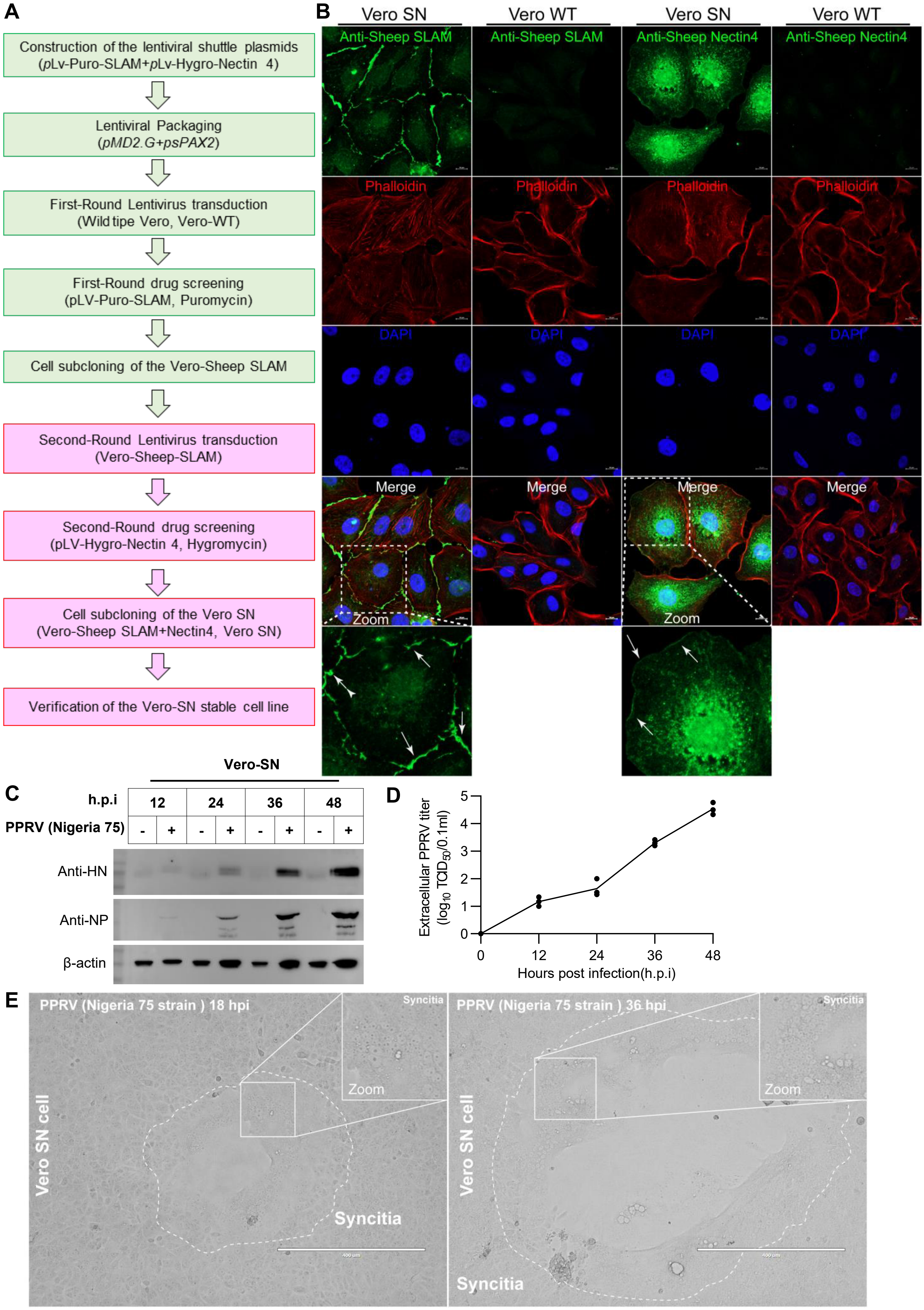

A
Construction of the lentiviral shuttle plasmids (pLv-Puro-SLAM+pLv-Hygro-Nectin 4)
Lentiviral Packaging (pMD2.G+psPAX2)
First-Round Lentivirus transduction (Wild tipe Vero, Vero-WT)
First-Round drug screening (pLV-Puro-SLAM, Puromycin)
Cell subcloning of the Vero-Sheep SLAM
Second-Round Lentivirus transduction (Vero-Sheep-SLAM)
Second-Round drug screening (pLV-Hygro-Nectin 4, Hygromycin)
Cell subcloning of the Vero SN (Vero-Sheep SLAM+Nectin4, Vero SN)
Verification of the Vero-SN stable cell line
B
Vero SN
Vero WT
Vero SN
Vero WT
Anti-Sheep SLAM
Anti-Sheep SLAM
Anti-Sheep Nectin4
Anti-Sheep Nectin4
Phalloidin
DAPI
Merge
Zoom
C
Vero-SN
h.p.i
12
24
36
48
PPRV (Nigeria 75)
- + - + - + - +
Anti-HN
Anti-NP
β-actin
D
Extracellular PPRV titer
(log10 TCID50/0.1ml)
0 1 2 3 4 5
0 12 24 36 48
Hours post infection(h.p.i)
E
PPRV (Nigeria 75 strain) 18 hpi
PPRV (Nigeria 75 strain) 36 hpi
Syncitia
Zoom
Vero SN cell
Syncitia
