# Supplementary material for: A novel monoclonal antibody targeting the hemagglutinin–neuraminidase of peste des petits ruminants virus maintains neutralizing activity by blocking viral adsorption and receptor interaction
Source: J Virol. 2026 Jun 26;100(7):e00787-26. doi: 10.1128/jvi.00787-26 (PMC13386946; doi:10.1128/jvi.00787-26)
Supplement: Supplemental legends — Fig. S1-S8 and Tables S1-S2. [file jvi.00787-26-s0009.docx]

**Supplemental figure legends**

**FIG S1. Preparation of the monoclonal antibody (mAb) targeting the PPRV HN protein.**

**(A)** Schematic representation of traditional hybridoma technology. The flowchart was created using the online BioRender website (BioRender.com).

**(B)** PCR amplification of the truncated HN gene derived from the virulent PPRV strain.

**(C)** Prokaryotic expression, purification, and identification of the PPRV HN protein.

WB analysis was performed to detect the His-tagged PPRV HN protein, as described in the Materials and Methods.

**(D)** Titer determination of the purified HN^1D4-4D9^. The hybridoma cell line (1D4-4D9) was propagated for antibody production. Subsequently, the hybridoma cells were inoculated into the mice's abdominal cavities to produce ascites. The collected ascitic fluid was further purified and analyzed using WB, as described in the Materials and Methods.

**FIG S2. Epitope mapping of the native state of the PPRV HN protein recognized by HN^1D4-4D9^.**

**(A and C)** Schematic representation and dominant epitope prediction for the first and second rounds of truncation during plasmid construction. Different colors and underlining indicate the positive groups used for mapping linear and conformational epitopes in the first and second rounds of truncation. B-cell epitopes were predicted using the online IEDB analysis resource (http://tools.iedb.org/bcell/).

**(B)** IFA analysis of the first-round truncation of PPRV HN. The plasmids, including pCAGGS, F1-pCAGGS (1-300aa)-HA, F2-pCAGGS (301-609aa)-HA, and F3-pCAGGS (200-440aa)-HA, were transfected into Vero cells. After 24 h post-transfection, the cells were prepared for IFA analysis. The red square border around the F2 fragments indicates the positive group for mapping conformational epitopes in the first-round of truncation.

**(D)** IFA analysis of the second-round truncation of PPRV HN. The plasmids, including pCAGGS, F4-pCAGGS (301-350aa)-HA, F5-pCAGGS (351-410aa)-HA, and F6-pCAGGS (411-440aa)-HA, were transfected into Vero cells. The red square border for the F5 fragments indicates the positive group for mapping conformational epitopes in the second-round of truncation.

**(E)** IFA analysis of the third- and fourth-round truncation of PPRV HN. The plasmids, including pCAGGS, F7-pCAGGS (351-390aa)-HA, F8-pCAGGS (351-400aa)-HA, F9-pCAGGS (200-388aa)-HA, F10-pCAGGS (200-387aa)-HA, F11-pCAGGS (200-386aa)-HA, F15-pCAGGS (360-440aa)-HA, F16-pCAGGS (370-440aa)-HA, F17-pCAGGS (373-440aa)-HA, F18-pCAGGS (375-440aa)-HA, F19-pCAGGS (377-440aa)-HA, F20-pCAGGS (379-609aa)-HA, F24-pCAGGS (383-609aa)-HA, or F25-pCAGGS (384-609aa)-HA, were transfected into Vero cells. The red square border around the F7 fragments indicates the positive group for mapping conformational epitopes in the third round of truncation.

**FIG S3. Epitope mapping of the denatured PPRV HN protein recognized by HN^1D4-4D9^.**

**(A, B, C, D, E, and F)** WB analysis of truncated HN fragments for linear epitope mapping in the first, second, third, and fourth rounds of truncation. Plasmids, including pCAGGS, pCAGGS-V-HN-HA, F1-pCAGGS (1-300aa)-HA, F2-pCAGGS (301-609aa)-HA, F3-pCAGGS (200-440aa)-HA, F4-pCAGGS (301-350aa)-HA, F5-pCAGGS (351-410aa)-HA, F6-pCAGGS (411-440aa)-HA, F7-pCAGGS (351-390aa)-HA, F8-pCAGGS (351-400aa)-HA, F12-pCAGGS (200-385aa)-HA, F13-pCAGGS (200-384aa)-HA, F15-pCAGGS (360-440aa)-HA, F16-pCAGGS (370-440aa)-HA, F17-pCAGGS (373-440aa)-HA, F18-pCAGGS (375-440aa)-HA, and F19-pCAGGS (377-440aa)-HA, were transfected into 293T cells. After 24 h post-transfection, cell samples were prepared for WB analysis, as described in the Methods and Materials section. The virulent PPRV strain is abbreviated as V. The underlining of F2, F5, and F7 indicates the positive groups for mapping linear and conformational epitopes in the first, second, third, and fourth rounds of truncation.

**FIG S4. Spatial structural prediction and simulation of HN proteins of virulent and attenuated PPRV strains.**

The nucleotide sequences of virulent and attenuated PPRV were translated into amino acid sequences. These sequences were submitted to the AlphaFold Protein Structure Database and saved in the PDB format. Subsequently, structure simulation and visualization were performed using the open-source PyMOL software. The key epitope residues of HN are shown in blue squares.

**FIG S5. Establishment of a Vero cell line stably expressing SLAM and nectin-4 receptors.**

**(A)** Schematic representation of Vero-SN cell line construction using a lentiviral packaging system.

**(B)** IFA analysis of the Vero-SN cell line. Wild-type Vero and Vero-SN cells were prepared for IFA analyses, as described in the Materials and Methods section. Phalloidin (red), SLAM/nectin-4 (green), and nuclei (blue).

**(C)** WB analysis of intracellular replication of PPRV in Vero-SN cells. Vero-SN cells were infected with attenuated PPRV (Nigeria 75/1) (TCID_50_ = 10^4.806^) at an MOI of 1. After 12, 24, 36, and 48 h post-infection, cell samples were collected for WB analysis.

**(D)** Determination of TCID_50_ for extracellular replication of PPRV in Vero-SN cells. Vero-SN cells were infected with attenuated PPRV (Nigeria 75/1) (TCID_50_ = 10^4.806^) at an MOI of 1. After 12, 24, 36, and 48 h post-infection, cell supernatant samples were collected for TCID_50_ analysis.

**(E)** Syncytium formation triggered by PPRV infection in the Vero-SN cell model. Vero-SN cells were infected with attenuated PPRV (Nigeria 75/1) (TCID_50_ = 10^4.806^) at an MOI of 1. At 18 and 36 h post-infection, typical syncytial images were captured using an EVOS M5000 imaging system.

**FIG S6. Comparative analysis of PPRV infectivity in wild-type Vero and Vero-SN cells.**

**(A)** Immunofluorescence observation of EGFP-PPRV infection. WT-Vero and Vero-SN cells were infected with attenuated EGFP-PPRV virions (TCID_50_ = 10^4.614^) at an MOI of 1. After 36 and 48 h of infection, representative images were captured using an EVOS M5000 imaging system. Wild-type Vero, WT-Vero.

**(B)** WB analysis of intracellular replication of PPRV infection. WT-Vero and Vero-SN cells were infected with attenuated PPRV (Nigeria 75/1) (TCID_50_ = 10^4.806^) at an MOI of 1. After 36 and 48 h of infection, cell samples were collected for WB analysis. The intensity band ratio of HN or NP to β-actin was quantified using ImageJ software.

**(C)** Determination of TCID_50_ for extracellular replication of PPRV infection. WT-Vero and Vero-SN cells were infected with attenuated PPRV (Nigeria 75/1) (TCID_50_ = 10^4.806^) at an MOI of 1. Cell supernatant samples were collected 36 and 48 h post-infection for TCID_50_ analysis.

**FIG S7. Observation of potent neutralizing activity of HN1D4-4D9 against PPRV in the Vero-SN cell model.**

**(A, B, and C)** Fluorescence and syncytium formation observations of the neutralizing activity of HN^1D4-4D9^ against EGFP-PPRV. Schematic timeline of the neutralization assay used to evaluate HN^1D4-4D9^ against PPRV infection. The diagram illustrates the experimental workflow over time (x-axis) for the three HN^1D4-4D9^ treatment strategies in response to extracellular mature EGFP-PPRV virions (TCID_50_ = 10^4.614^) at an MOI of 1: pre-, post-, and simultaneous mixture treatments. The key procedural steps are color-coded. Blue: Treatment of Vero-SN cells or virus with HN^1D4-4D9^; Green: Exposure to extracellular mature PPRV virions (EGFP-PPRV); Red: Critical wash with PBS to remove unbound, non-internalized virions after viral adsorption. At 24, 48, and 72 h post-infection, immunofluorescence intensity and syncytia formation were assessed. Representative images were captured using an EVOS M5000 imaging system.

**FIG S8. Molecular docking and dynamic simulations of the PPRV HN protein with cellular receptors.**

**(A)** Molecular docking simulations of the PPRV HN protein with the cellular receptors. The nucleotide sequences of sheep-derived SLAM, sheep-derived nectin-4, and full-length and epitope-deleted HN of virulent PPRV strains were translated into amino acid sequences and submitted to the AlphaFold 3 protein structure database, and the resulting structures were saved in PDB format. Subsequently, structural simulations, visualizations, and molecular docking were performed using PyMOL 2.5 (https://pymol.org/2/). Full-length HN and epitope-deleted HN (purple); SLAM and nectin-4 (green); epitopes (blue).

**(B)** Time-dependent root-mean-square deviation (RMSD) of full-length HN–SLAM and epitope-deleted HN–SLAM complexes over 10 ns of molecular dynamics simulations. Structural dynamics simulations were performed using GROMACS (<https://www.gromacs.org/>). The epitope-deleted HN (HN-Δ381-387aa) – SLAM complex exhibited RMSD values comparable to or slightly lower than those of the full-length counterpart.

**(C)** Time-dependent RMSD of full-length HN–nectin4 and epitope-deleted HN–Nectin4 complexes throughout 10 ns of simulations. Structural dynamics simulations were performed using GROMACS (<https://www.gromacs.org/>). Neither complex displayed a sustained upward trend or pronounced large-scale oscillations, indicating that epitope deletion does not destabilize the HN–Nectin4 interaction. Notably, the RMSD fluctuations of the epitope-deleted HN (HN-Δ381-387aa)–Nectin4 complex were somewhat reduced during the later phase.

**Table legends**

**Table S1. Primer sequences used to construct the truncated plasmid of PPRV HN in this study.**

| Name | Primer sequence (5'→3') | Length(bp) |
| --- | --- | --- |
| F1 (1-300aa)-F | catcattttggcaaaGAATTCGCCACCatgtccgcacaaagggagag | 900 |
| F1 (1-300aa)-R | gctcgagcatgcccgGGTACCTTAagcgtaatctggaacatcgtatgggtaGCTTCCTCCTCCGCATAGGGCTGTCAACTTC |  |
| F2 (301-609aa)-F | catcattttggcaaaGAATTCGCCACCATGACCTCATCTGAGACTGTG | 927 |
| F2 (301-609aa)-R | gctcgagcatgcccgGGTACCTTAagcgtaatctggaacatcgtatgggtaGCTTCCTCCTCCgACTGGATTACATGTTAC |  |
| F3 (200-440aa)-F | catcattttggcaaaGAATTCGCCACCATGgaacttacaatgaccttaatgg | 723 |
| F3 (200-440aa)-R | gctcgagcatgcccgGGTACCTTAagcgtaatctggaacatcgtatgggtaGCTTCCTCCTCCgccggataggtgaggtatc |  |
| F4 (301-350aa)-F | catcattttggcaaaGAATTCGCCACCATGACCTCATCTGAGACTGTG | 150 |
| F4 (301-350aa)-R | gctcgagcatgcccgGGTACCTTAagcgtaatctggaacatcgtatgggtaGCTTCCTCCTCCATAGAGTTTCTCCACCATGAGATCAG |  |
| F5 (351-410aa)-F | catcattttggcaaaGAATTCGCCACCATGTTgTCTTCACATAGAGGGATcATCAAaG | 180 |
| F5 (351-410aa)-R | gctcgagcatgcccgGGTACCTTAagcgtaatctggaacatcgtatgggtaGCTTCCTCCTCCgtaggcagggattctcccttctgacc |  |
| F6 (411-440aa)-F | catcattttggcaaaGAATTCGCCACCATGggggtgatcagggtcagtcttgac | 90 |
| F6 (411-440aa)-R | gctcgagcatgcccgGGTACCTTAagcgtaatctggaacatcgtatgggtaGCTTCCTCCTCCgccggataggtgaggtatcagtg |  |
| F7 (351-390aa)-F | catcattttggcaaaGAATTCGCCACCATGTTgTCTTCACATAGAGGGATcATCAAaG | 120 |
| F7 (351-390aa)-R | gctcgagcatgcccgGGTACCTTAagcgtaatctggaacatcgtatgggtaGCTTCCTCCTCCaggtcgagtcttgcatgcttccacc |  |
| F8 (351-400aa)-F | catcattttggcaaaGAATTCGCCACCATGTTgTCTTCACATAGAGGGATcATCAAaG | 150 |
| F8 (351-400aa)-R | gctcgagcatgcccgGGTACCTTAagcgtaatctggaacatcgtatgggtaGCTTCCTCCTCCgcctgatcctgtgccattgcaaaatg |  |
| F9 (200-388aa)-F | catcattttggcaaaGAATTCGCCACCATGgaacttacaatgaccttaatgg | 567 |
| F9 (200-388aa)-R | gctcgagcatgcccgGGTACCTTAagcgtaatctggaacatcgtatgggtaGCTTCCTCCTCCagtcttgcatgcttccaccag |  |
| F10 (200-387aa)-F | catcattttggcaaaGAATTCGCCACCATGgaacttacaatgaccttaatgg | 564 |
| F10 (200-387aa)-R | gctcgagcatgcccgGGTACCTTAagcgtaatctggaacatcgtatgggtaGCTTCCTCCTCCcttgcatgcttccaccagac |  |
| F11 (200-386aa)-F | catcattttggcaaaGAATTCGCCACCATGgaacttacaatgaccttaatgg | 561 |
| F11 (200-386aa)-R | gctcgagcatgcccgGGTACCTTAagcgtaatctggaacatcgtatgggtaGCTTCCTCCTCCgcatgcttccaccagacattc |  |
| F12 (200-385aa)-F | catcattttggcaaaGAATTCGCCACCATGgaacttacaatgaccttaatgg | 558 |
| F12 (200-385aa)-R | gctcgagcatgcccgGGTACCTTAagcgtaatctggaacatcgtatgggtaGCTTCCTCCTCCtgcttccaccagacattcacc |  |
| F13 (200-384aa)-F | catcattttggcaaaGAATTCGCCACCATGgaacttacaatgaccttaatgg | 555 |
| F13 (200-384aa)-R | gctcgagcatgcccgGGTACCTTAagcgtaatctggaacatcgtatgggtaGCTTCCTCCTCCttccaccagacattcacctttattc |  |
| F14 (200-383aa)-F | catcattttggcaaaGAATTCGCCACCATGgaacttacaatgaccttaatgg | 552 |
| F14 (200-383aa)-R | gctcgagcatgcccgGGTACCTTAagcgtaatctggaacatcgtatgggtaGCTTCCTCCTCCcaccagacattcacctttattctg |  |
| F15 (360-440a)-F | catcattttggcaaaGAATTCGCCACCATGGATGAtGAGGCCAATTGGGTAGTGccg | 243 |
| F15 (360-440aa)-R | gctcgagcatgcccgGGTACCTTAagcgtaatctggaacatcgtatgggtaGCTTCCTCCTCCgccggataggtgaggtatc |  |
| F16 (370-440aa)-F | catcattttggcaaaGAATTCGCCACCATGaccgatgttcgtgatcttcagaataaagg | 213 |
| F16 (370-440aa)-R | gctcgagcatgcccgGGTACCTTAagcgtaatctggaacatcgtatgggtaGCTTCCTCCTCCgccggataggtgaggtatc |  |
| F17 (373-440aa)-F | catcattttggcaaaGAATTCGCCACCATGcgtgatcttcagaataaaggtgaatg | 204 |
| F17 (373-440aa)-R | gctcgagcatgcccgGGTACCTTAagcgtaatctggaacatcgtatgggtaGCTTCCTCCTCCgccggataggtgaggtatc |  |
| F18 (375-440aa)-F | catcattttggcaaaGAATTCGCCACCATGcttcagaataaaggtgaatgtctggtgg | 198 |
| F18 (375-440aa)-R | gctcgagcatgcccgGGTACCTTAagcgtaatctggaacatcgtatgggtaGCTTCCTCCTCCgccggataggtgaggtatc |  |
| F19 (377-440aa)-F | catcattttggcaaaGAATTCGCCACCATGaataaaggtgaatgtctggtggaagcatg | 192 |
| F19 (377-440aa)-R | gctcgagcatgcccgGGTACCTTAagcgtaatctggaacatcgtatgggtaGCTTCCTCCTCCgccggataggtgaggtatc |  |
| F20 (379-609aa)-F | catcattttggcaaaGAATTCGCCACCATGggtgaatgtctggtggaagcatgcaag | 693 |
| F20 (379-609aa)-R | gctcgagcatgcccgGGTACCTTAagcgtaatctggaacatcgtatgggtaGCTTCCTCCTCCgACTGGATTACATGTTAC |  |
| F21 (380-609aa)-F | catcattttggcaaaGAATTCGCCACCATGgaatgtctggtggaagcatgcaagactc | 690 |
| F21 (380-609aa)-R | gctcgagcatgcccgGGTACCTTAagcgtaatctggaacatcgtatgggtaGCTTCCTCCTCCgACTGGATTACATGTTAC |  |
| F22 (381-609aa)-F | catcattttggcaaaGAATTCGCCACCATGtgtctggtggaagcatgcaagactcgac | 687 |
| F22 (381-609aa)-R | gctcgagcatgcccgGGTACCTTAagcgtaatctggaacatcgtatgggtaGCTTCCTCCTCCgACTGGATTACATGTTAC |  |
| F23 (382-609aa)-F | catcattttggcaaaGAATTCGCCACCATGctggtggaagcatgcaagactcgacctc | 684 |
| F23 (382-609aa)-R | gctcgagcatgcccgGGTACCTTAagcgtaatctggaacatcgtatgggtaGCTTCCTCCTCCgACTGGATTACATGTTAC |  |
| F24 (383-609aa)-F | catcattttggcaaaGAATTCGCCACCATGgtggaagcatgcaagactcgacctccttc | 681 |
| F24 (383-609aa)-R | gctcgagcatgcccgGGTACCTTAagcgtaatctggaacatcgtatgggtaGCTTCCTCCTCCgACTGGATTACATGTTAC |  |
| F25 (384-609aa)-F | catcattttggcaaaGAATTCGCCACCATGgaagcatgcaagactcgacctccttc | 678 |
| F25 (384-609aa)-R | gctcgagcatgcccgGGTACCTTAagcgtaatctggaacatcgtatgggtaGCTTCCTCCTCCgACTGGATTACATGTTAC |  |
| V-hn (Δ381-387aa)-F | gaataaaggtgaaactcgacctccttcattttgcaatg | 1806 |
| V-hn (Δ381-387aa)-R | gaggtcgagtttcacctttattctgaagatcacgaac |  |
| F3 (Δ381-387aa)-F | gaataaaggtgaaactcgacctccttcattttgcaatg | 702 |
| F3 (Δ381-387aa)-R | gaggtcgagtttcacctttattctgaagatcacgaac |  |
| V-hn (Δ380-385aa)-F | gaataaaggttgcaagactcgacctccttcattttgcaatggc | 1809 |
| V-hn (Δ380-385aa)-R | cgagtcttgcaacctttattctgaagatcacgaacatcggtag |  |
| F3 (Δ380-385aa)-F | gaataaaggttgcaagactcgacctccttcattttgcaatggc | 705 |
| F3 (Δ380-385aa)-R | cgagtcttgcaacctttattctgaagatcacgaacatcggtag |  |

**Table S2. Primer sequences used to construct the mutated plasmids of PPRV HN in this study.**

| Name | Primer sequence (5'→3') |
| --- | --- |
| Hn (E380A)-F | gaataaaggtgcttgtctggtggaagcatgcaagac |
| Hn (E380A)-R | caccagacaagcacctttattctgaagatcacgaacatc |
| Hn (C381A)-F | gaataaaggtgaagctctggtggaagcatgcaag |
| Hn (C381A)-R | ccagagcttcacctttattctgaagatcacgaacatc |
| Hn (L382A)-F | gtgaatgtgctgtggaagcatgcaagactcg |
| Hn (L382A)-R | cttccacagcacattcacctttattctgaagatcacg |
| Hn (V383A)-F | tgtctggctgaagcatgcaagactcgac |
| Hn (V383A)-R | gcatgcttcagccagacattcacctttattctg |
| Hn (E384A)-F | tggtggctgcatgcaagactcgacctc |
| Hn (E384A)-R | gcatgcagccaccagacattcacctttattctg |
| Hn (A385A)-F | ggtggaagcttgcaagactcgacctccttc |
| Hn (A385A)-R | gtcttgcaagcttccaccagacattcacctttattc |
| Hn (C386A)-F | gaagcagctaagactcgacctccttcattttgc |
| Hn (C386A)-R | gagtcttagctgcttccaccagacattcacc |
| Hn (K387A)-F | gcatgcgctactcgacctccttcattttgcaatg |
| Hn (K387A)-R | gtcgagtagcgcatgcttccaccagacattc |
